# Integration of metabolites from meta-analysis with transcriptome reveals enhanced *SPHK1* in PDAC with a background of pancreatitis

**DOI:** 10.1186/s12885-022-09816-6

**Published:** 2022-07-19

**Authors:** Vijayasarathy Ketavarapu, Vishnubhotla Ravikanth, Mitnala Sasikala, G. V. Rao, Ch. Venkataramana Devi, Prabhakar Sripadi, Murali Satyanarayana Bethu, Ramars Amanchy, H. V. V. Murthy, Stephen J. Pandol, D. Nageshwar Reddy

**Affiliations:** 1 Asian Healthcare Foundation, Asian Institute of Gastroenterology, Mindspace Rd, Gachibowli, Hyderabad, Telangana 500032 India; 2 AIG Hospitals, Mindspace Rd, Gachibowli, Hyderabad, Telangana 500032 India; 3 Department of Biochemistry, University College of Science, Osmania University, Hyderabad, 500 007 India; 4 Centre for Mass Spectrometry, Analytical & Structural Chemistry Department, CSIR-Indian Institute of Chemical Technology, Tarnaka, Hyderabad, 500 007 India; 5 Division of Applied Biology, CSIR-IICT (Indian Institute of Chemical Technology), Ministry of Science and Technology (GOI), Hyderabad, Telangana 500007 India; 6 Department of Pharmacology and Therapeutics, Roswell Park Comprehensive Cancer Center, Elm &Carlton Streets, Buffalo, New York, 14221 USA; 7 Department of Medicine, Division of Digestive and Liver Diseases, Cedars-Sinai Medical Center, Los Angeles, CA USA

**Keywords:** Chronic pancreatitis, Pancreatic ductal adenocarcinoma, PDAC with background of CP, SPHK1, Transcriptomics, Metabolomic biomarkers

## Abstract

**Background:**

Pathophysiology of transformation of inflammatory lesions in chronic pancreatitis (CP) to pancreatic ductal adenocarcinoma (PDAC) is not clear.

**Methods:**

We conducted a systematic review, meta-analysis of circulating metabolites, integrated this data with transcriptome analysis of human pancreatic tissues and validated using immunohistochemistry. Our aim was to establish biomarker signatures for early malignant transformation in patients with underlying CP and identify therapeutic targets.

**Results:**

Analysis of 19 studies revealed AUC of 0.86 (95% CI 0.81-0.91, *P <* 0.0001) for all the altered metabolites (*n =* 88). Among them, lipids showed higher differentiating efficacy between PDAC and CP; *P-*value (< 0.0001). Pathway enrichment analysis identified sphingomyelin metabolism (impact value-0.29, FDR of 0.45) and TCA cycle (impact value-0.18, FDR of 0.06) to be prominent pathways in differentiating PDAC from CP. Mapping circulating metabolites to corresponding genes revealed 517 altered genes. Integration of these genes with transcriptome data of CP and PDAC with a background of CP (PDAC-CP) identified three upregulated genes; *PIGC*, PPIB, *PKM* and three downregulated genes; *AZGP1*, *EGLN1*, *GNMT*. Comparison of CP to PDAC-CP and PDAC-CP to PDAC identified upregulation of *SPHK1*, a known oncogene.

**Conclusions:**

Our analysis suggests plausible role for *SPHK1* in development of pancreatic adenocarcinoma in long standing CP patients. *SPHK1* could be further explored as diagnostic and potential therapeutic target.

**Supplementary Information:**

The online version contains supplementary material available at 10.1186/s12885-022-09816-6.

## Introduction

Pancreatic adenocarcinoma of ductal origin is reported to be the third leading cause of cancer related mortality [1]. Chronic pancreatitis (CP) poses 2-3 fold higher risk of developing PDAC in comparison to general population [2, 3]. However, very little is known about the malignant transformation of CP to PDAC. Hence an urgent need arises for studying complex networks of exocrine disease of pancreas progressing to pancreatic cancer. Most of the times patients present at advanced stage of the disease with a poor prognosis and overall survival rate less than 10% [4]. Identification of accurate biomarkers during the progression of acute to chronic pancreatitis and to various stages of pancreatic cancer (PDAC) [5, 6] is still a highly coveted goal. Metabolomics approach aids in assessing the metabolic alterations to reveal mechanisms of metabolic stress during CP to PDAC transformation and design new diagnostic and therapeutic approaches [7]. In this study, we performed systematic review and meta-analysis of circulatory metabolite biomarkers in CP and PDAC. In addition, we chose to integrate the genes corresponding to altered metabolites with our experimental transcriptome data in CP, PDAC and PDAC-CP to identify genes and major metabolic pathways that are likely involved in malignant transformation of CP.

### Study design

In order to achieve our goal, we first identified metabolomics studies (*n =* 25) in CP and PDAC, performed meta-analysis and identified metabolic networks using metaboAnalyst. Then, our aim was to confirm the metabolite alterations at the level of corresponding gene expression. Therefore, we retrieved genes corresponding to altered metabolites employing relational databases and integrated with dysregulated gene datasets that were extracted from transcriptomes in CP, PDAC-CPand PDAC. To validate our findings, immunohistochemistry was performed on pancreatic tissues from CP, PDAC-CP and PDAC patients.

## Materials and methods

All authors had access to the study data, reviewed and approved the final manuscript. Systematic review and meta-analysis were conducted using a predefined protocol (Additional file 1: Table S1) as recommended by MOOSE guidelines [8].

### Systematic literature search

**Fig. 1:**
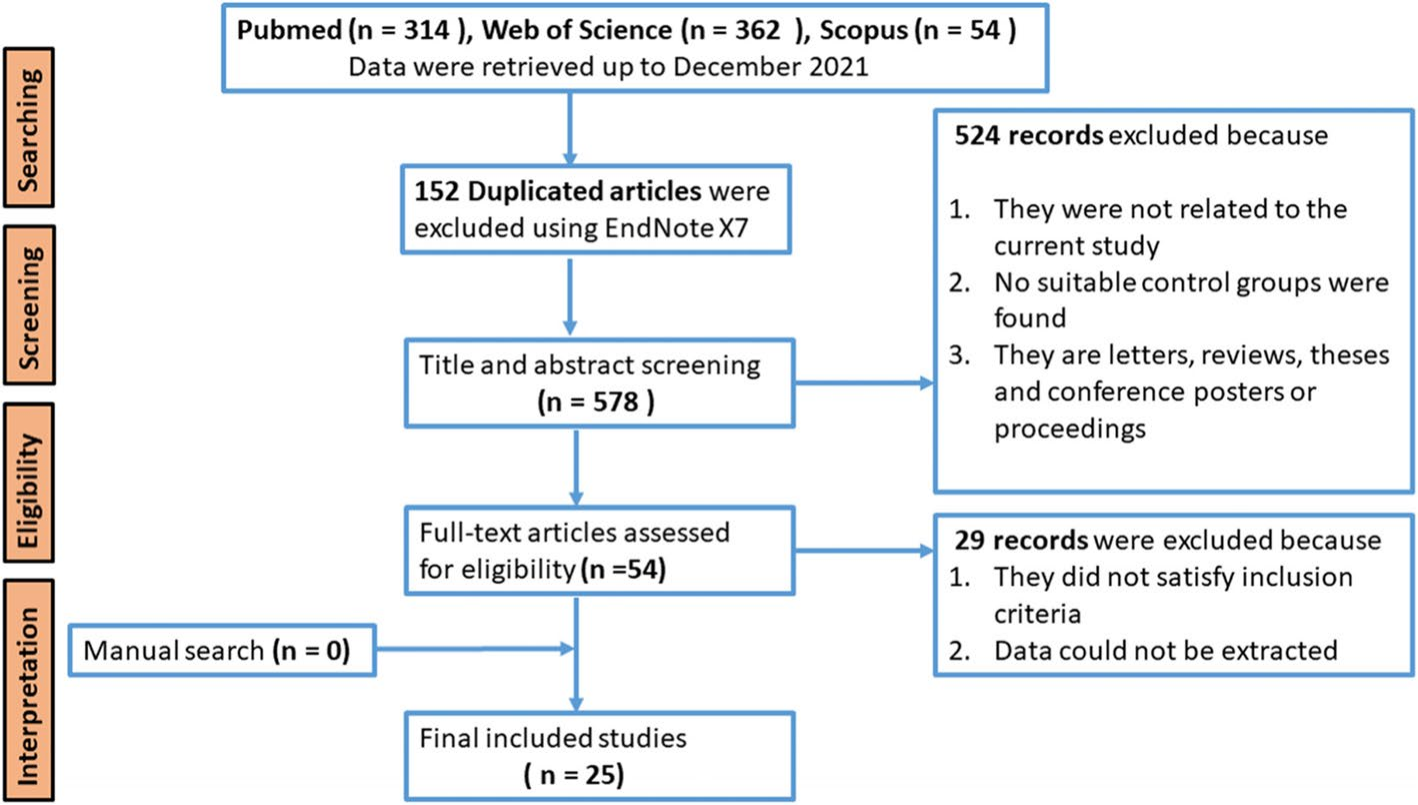
Schematic flowchart of systematic review

A systematic search of PubMed, Web of Science and Scopus, was carried out to identify biomarker studies that reported metabolites in CP and PDAC (Fig. 1). The following terms were applied to search criteria: “pancreas or pancreatic” and “cancer or carcinoma or chronic pancreatitis” and “metabolite profiling or metabolomics or metabolome or metabolite analysis or metabolic characterization” and “serum or plasma or biomarkers or markers”. The literature search was conducted from March 2005 till December 2021. The references that were found relevant from letters, thesis and posters presented during conferences were reviewed for further analysis.

### Inclusion and exclusion criteria

Title and year of publications, authors and abstracts were extracted to EndNote X7(Thomson Reuters, NY, USA). Following redundancy check, an initial screening process was employed to screen the titles and abstracts of the eligible articles. Articles found relevant were further selected for reading. Authors have been advised to avoid bias and a consensus was made in article selection. Only studies that examined and validated using metabolomic platforms in CP and PDAC especially in serum or plasma-based marker discovery were eligible. Studies included the following criteria: (1) Biomarker in circulation (serum / plasma); (2) metabolomics platform (GC/MS, LC/MS, NMR); (3) biomarker selection was selected based on statistical significance and further evaluation by statistical learning for discrimination of PDAC from healthy control or CP; (4) accuracy, sensitivity, specificity and area under the curve (AUC-ROC, etc.) were deemed as suitable prediction metrics. Exclusion of studies was followed if (1) no suitable control groups were used; (2) the studies were conducted on animal tissues, cell lines and urine samples; (3) there was duplication of data and (4) they are letters, reviews, theses, and conference proceedings.

### Study data extraction

The data were abstracted by at least two independent researchers. A data extraction form was made in accordance with earlier systematic review guidelines [9]. For preliminary analysis, information pertaining to the study including first author, year of publication, patient/control characteristics (age, gender, clinical stage) were extracted. The nature of biological specimen, platforms employed for analysis, proper targeted / untargeted approach, sample collection, storage, sample preparation, pretreatment description, analytical standards, validation and metabolite identification methods were described. The list of biomarker panels were extracted. For each biomarker study comprising controls, CP and PDAC, fold changes values were noted or the biomarker levels were grouped as elevated or low respectively. Total patients for each study along with the biomarker panel, area under curve, sensitivity and specificity values were noted. Following extraction of biomarker data, a third independent researcher reviewed abstracted data. Data were discussed and sorted in case of discrepancy.

### Outcome measures

The study outcome measures are

1. Altered metabolite levels in CP and PDAC
2. Subgrouping of metabolites for efficient differentiation of CP and PDAC
3. Identification of metabolic pathways
4. Integration of corresponding genes of altered metabolites with pancreatic transcriptome to identify genes and major metabolic pathways with potential involvement in malignant transformation.

### Reporting methodological quality assessment

#### Metabolomics standards initiative

prescribes gold standards in metabolomic experiments, sample preparation, experimental analysis, quality control, metabolite identification, and data pre-processing [10]. General guidelines were followed for evaluation of a biomarker study, defined objective, definitive clinical question, reliable data source, significant statistical analysis, and secondary validation [11, 12]. We developed a quality reporting panel to fully assess the criteria in the selected studies with (1) reference standards (e.g., CA19.9 for pancreatic cancer/ PDAC) (2) the sampling and experimental procedures, metabolite identification and (3) the bioinformatic analysis including data mining, parsing, statistical analysis, and modeling using HMDB database. Meta-analysis was carried out using MedCalc statistical software (ver. 19.2.6) for all publications which reported AUC, sensitivity by deriving standard error. To assess the risk of bias, publication bias was carried out for publications which reported standard error and AUC values using MedCalc software [13].

### Statistical and pathway enrichment analysis

Data pertaining to 25 biomarker studies data were identified and entered into MS-excel. Meta-analysis was performed using MedCalc software for all publications which reported AUC, specificity, sensitivity by deriving standard error. We computed the standard error from samples size(n) for each study from AUC and then grouped the studies as all metabolites, lipids, carbohydrates and amino acids. Using a two tailed critical value of normal distribution, and keeping sample size between two groups we computed AUC, the standard error and 95% confidence interval. AUC and standard error were used to generate the forest plots for all metabolite markers, carbohydrate, amino acid and lipid metabolites. Assessment of statistical heterogeneity by forest plots and publication bias were by funnel plot asymmetry were performed using Egger’s intercept. The compound name standardization was followed using Human Metabolome Database version 4.0 [14]. The pathway analysis was conducted using metaboAnalyst (Ver5) [15]. The Kyoto Encyclopedia of Genes and Genomes (KEGG) and the small molecule pathway (SMP) were used to identify pathways of significance to the study [16]. The analysis algorithm was a hypergeometric test for overrepresentation analysis, and the relative betweenness centrality was selected for pathway topology analysis. Pathways that had an adjusted *P-*value (false discovery rate, FDR) of less than 0.05 were considered to be significantly enriched. Subgroup analysis was screened by metaboAnalyst [17] and overall mapping pathways were derived for metabolic intermediates and networks of significance to differentiate healthy controls from PDAC, healthy controls/CP from PDAC and CP from PDAC.

### Transcriptome analysis and gene ontology

For integrating the genes of altered metabolites identified from meta-analysis, transcriptome profiles were generated in pancreatic tissues with CP, PDAC-CP, PDAC and healthy tissue. Paraffin embedded tissue blocks (FFPE) were retrieved from the archived samples. A total of 12 tissues were included of which 3 were normal pancreatic tissues (from trauma cases), 3 with CP, 4 with PDAC-CP and 2 with PDAC. All the participants provided written informed consent and the study protocol conformed to the ethical guidelines of the 1975 Declaration of Helsinki. The study was approved by the Institutional Review Board (Protocol #AIG/AHF-IRB:02/31/20) of Asian Healthcare Foundation, a research wing of AIG hospitals. Transcriptome datasets are available online, (Vishnubhotla, Ravikanth (2021), “Transcriptome Dataset”, Mendeley Data, V1, doi: 10.17632/2jjbpvfm72.1). CP was diagnosed with the clinical symptoms: chronic abdominal pain, pancreatic calcifications, abdominal pain and morphological characteristics from duct pancreatograms and exocrine pancreatic insufficiency. PDAC was diagnosed by histopathologic examination. Patients who developed PDAC with long standing CP were considered as PDAC-CP (Additional file 1: Table S2). RNA was isolated from the FFPE blocks using RNeasy FFPE kit (Qiagen) as per manufacturer’s instructions. cRNA was synthesized and hybridized to Affymetrix Human Transcriptome arrays 2.0 and scanned. Transcriptome data was analyzed using Affymetrix Transcriptome Analysis Console 3.0.0.466. Transcriptome data is represented as s**plicing index** which is similar to the fold change (FC) that is often used on the gene level. The exon expression is first normalized to the expression of the corresponding gene before calculating the ratio between CP and PDAC-CP. Transcript level dysregulation were identified between the patient groups. GO enrichment analysis were performed using PANTHER and GO ontology database [18].

### Integration of genes of altered metabolites with transcriptome

Genes corresponding to the altered metabolites differentiating CP and PDAC were extracted from relational database of metabolic pathways [19] that integrates KEGG reactome [20] and HMDB [14]. We retrieved dysregulated gene list from the transcriptome data of CP and PDAC-CP generated from our laboratory. The two gene sets were compared to identify common and unique genes between CP and PDAC-CP with a likely role in malignant transformation. Additionally, dysregulated gene list from PDAC-CP was also compared to that of PDAC. TSGene 2.0 [21] database was compared to retrieve tumour suppressor genes from our identified gene datasets.

### Immunohistochemistry

To validate the findings of integrating meta-analysis of metabolome and in-house transcriptomic data, we performed immunohistochemistry on the paraffin embedded tissue sections of CP(*n =* 6), PDAC-CP (*n =* 5) and PDAC (*n =* 4) obtained from department of pathology archives. 4 um sections were stained with anti-human SPHK1-HRP antibody (mouse origin, Santacruz biotechnologies Inc., USA), CD31 antibody (mouse origin) using BioGenex detection system. Each tissue was observed using a light microscope at a total magnification of 40X and documented using a computer with cellSens imaging software and a camera that had been integrated with Olympus BX63 microscope. Photographs were taken randomly with a total of ten visual fields per one tissue for acini, blood vessel, duct, islet and standardized using a global white balance. The brown colour intensity was calculated using the plugin program in Image J, IHC profiler [22] which quantified the intensity of the images. The results of quantification were expressed as H-Score [23] based on the formula: (% low positive × 1) + (% positive × 2) + (% high positive × 3). Fisher’ exact test was used to compare SPHK1 expression among the two groups: CP, PDAC-CP and PDAC-CP, PDAC with an *α* of 0.05 as a cut-off to denote statistical significance.

## Results

### Systematic review

Studies were included from Web of Science, Scopus, PubMed and were screened for title, abstract and duplications were deleted. Inclusion and exclusion criteria were followed as described earlier. Letters, reviews, theses, conference posters or proceedings were also excluded. 54 studies were found to be eligible for further full-text assessment, and only 25 studies could be included for systematic review (Fig. 1).

### Characteristics of studies included

The characteristics of studies included are described in Additional file 2: Dataset S1. A total of 10,951 controls (either healthy controls or CP patients were considered as controls) and 2248 patients with PDAC, pancreatic cancer were included in the analysis (Additional file 2: Dataset S1). Healthy controls were used in 15 studies while 8 studies used either healthy controls or CP patients to compare to PDAC and 2 used CP as controls. Apart from one study [24] which included 240 PDAC and 7772 controls, the sample size of the remaining studies were relatively small, of which six studies out of 25 comprised more than 100 PDAC patients. Tumour stage was provided in 16 studies, out of which 6 studies included PDAC patients who were in resectable stages [25–30].

### Metabolomics design properties of included studies

The study design and metabolomic methods followed in the studies were tabulated (Additional file 2: Dataset S1). Untargeted metabolomics approach was followed by 17 studies (68%) while 2 studies used only lipidomics. Among studies that used targeted metabolomics, focus was on amino acids [24, 25, 31–33] and fatty acids [34]. Most preferred platform was Mass spectrometry based assessment of novel biomarkers (20 studies), followed by NMR (5 studies) [31, 35–38]. Eleven studies collected samples under fasting condition, and only two studies performed outlier detection, using Principal component analysis.

### Meta-analysis and publication bias

**Fig. 2:**
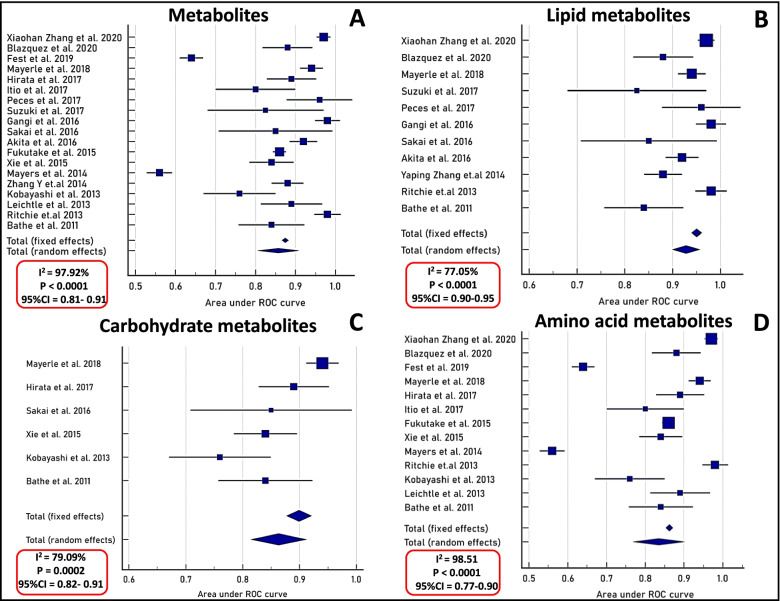
Forest plots of altered metabolites identified in meta-analysis (**A**) Forest plot of 19 metabolomic studies using AUC values and computed standard error. Heterogeneity (I^2^) was assessed for fixed and random effects (**B**) Forest plot of lipid metabolites retrieved from 11 studies (**C**) Forest plot of carbohydrate metabolites retrieved from 6 studies (**D**) Forest plot of amino acid metabolites retrieved from 13 studies. Metabolic pathway analysis using metaboAnalyst identified (**E**) Enriched glycerophospholipid pathway for circulatory metabolites detected in healthy control and PDAC. Metabolites marked in red are altered in PDAC patients as compared to healthy controls (**F**) metaboAnalyst analysis identified enriched arginine and glutamate metabolism for circulatory metabolites detected in PDAC as compared to healthy controls/chronic pancreatitis. Metabolites are marked in red and their fold changes are in blue color (**G**) metaboAnalyst analysis identified enriched sphingomyelin pathway and TCA cycle for circulatory metabolites detected in PDAC and CP patients. Metabolites are marked in red and their fold changes are in blue color

Studies included in the meta-analysis identified panels of lipids, carbohydrates and amino acids to differentiate CP from PDAC. The heterogeneity was more than 95% in all classes of biomarkers and all were statistically significant. Among the studies which reported AUC, heterogeneity was higher in amino acids followed by phospholipids and carbohydrates (Fig. 2). Heterogeneity was more than 90% when random effects meta-analysis was performed for studies reporting sensitivity and were statistically significant (*SI* Additional file 1: Fig. S1). A total of 19 metabolomic studies compared either healthy, CP and PDAC patients and reported AUC values. Standard error was calculated from AUC values for generating forest plots. For all the circulatory biomarkers detected, the AUC was observed to be 0.86 (95% confidence interval 0.81 to 0.91), with heterogeneity 97.92% using the random effects model (Fig. 2A). We further classified these biomarker studies to amino acid, carbohydrate and lipid metabolites. Compared to other metabolite biomarkers we observed that, lipid metabolites (Fig. 2B) differentiate PDAC from CP with higher AUC 0.93 (95% confidence interval 0.90 to 0.95 with heterogeneity 77%, *P-*value (< 0.0001) followed by amino acids with AUC 0.83(95% confidence interval 0.77 to 0.90 with heterogeneity 98.51%, *P-*value (< 0.0001) (Fig. 2D) and carbohydrates (Fig. 2C) with AUC 0.863 (95% confidence interval 0.82 to 0.91 with heterogeneity 79%, *P-*value (0.0002). Egger’s test and funnel plot (Additional file 3: Dataset S2; *p* value = 0.5) suggest no publication bias among the selected biomarker studies (Additional file 1: Fig. S1).

### Pathway enrichment analysis of potential biomarkers

**Fig. 3:**
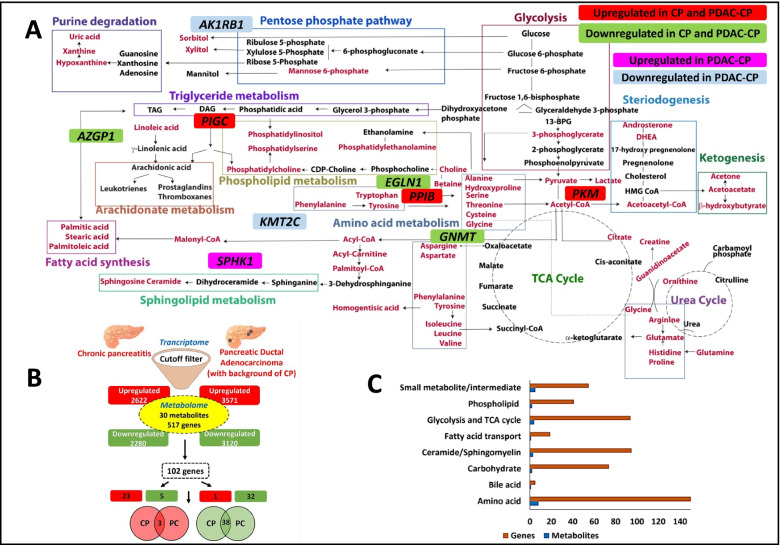
Metabolic networks, genes and integration of transcriptome (**A**) Metabolites mapped from selected studies (red) and their networks of significance operating in chronic pancreatitis and pancreatic cancer patients. PIGC, PPIB and PKM are upregulated in CP, PDAC-CP. AZGP1, EGLN1 and GNMT are downregulated in CP and PDAC-CP. SPHK1 upregulated in PDAC-CP and AK1RB1, KMT2C are downregulated in PDAC-CP. These genes are mapped to their respective metabolic pathways (**B**) Integration of metabolome and transcriptome yielding 102 common genes in chronic pancreatitis and pancreatic cancer human tissues (**C**) Distribution of genes and types of metabolites

To identify metabolic pathways differentiating PDAC from CP, we have categorized the studies into 3 groups; i) controls vs PDAC ii) controls/CP vs PDAC iii) CP vs PDAC. Glycerophospholipid metabolism with 45 metabolites (impact value of 0.22, FDR of 1) was the most prominent pathway in differentiating healthy controls from PDAC (Fig. 2E) Glutamate (impact value of 0.5, FDR of 1.36E-02) and arginine metabolism (impact value of 0.19, FDR of 3.45E-03) were the two prominent pathways derived from list of 13 biomarkers (Fig. 2F) differentiating healthy controls/CP from PDAC. Sphingomyelin pathway (impact value of 0.29, FDR of 0.45) and TCA cycle (impact value of 0.18, FDR of 0.06) were the two prominent pathways derived from a list of 30 biomarkers (Fig. 2G) (Additional file 4: Dataset S3) that differentiated CP from PDAC. We further grouped all the biomarkers from these studies and an overall metabolic flux among various intermediates was generated (Fig. 3A). A total of 517 genes corresponding to the 30 metabolites differentiating CP from PDAC were extracted from relational databases to integrate with transcriptome data sets (Additional file 5: Dataset S4).

### Transcriptome in CP, PDAC-CP and PDAC

**Fig. 4:**
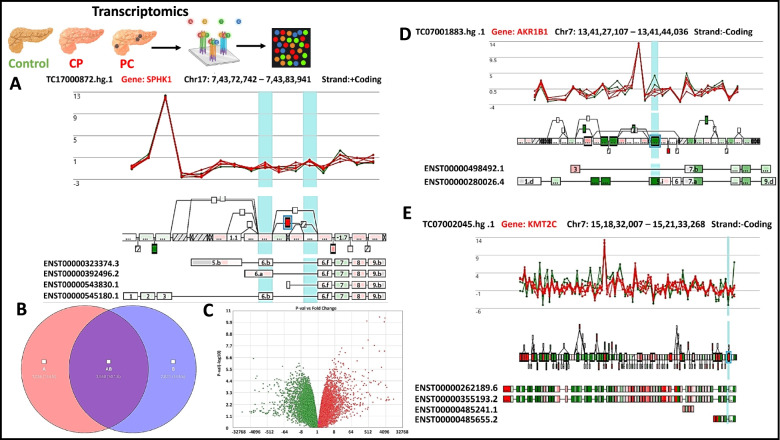
Transcriptomics (**A**) Splicing index of SPHK1(4.01), upregulated gene in PDAC-CP tissue, red and green lines represent splicing index between CP and PDAC-CP (**B**) Venn diagram of genes overlapped in CP and PDAC-CP tissues, genes unique in CP, 1553 genes (pink) 13.4%, unique in PDAC-CP, 4220 genes 36.5%(blue), overlapped between CP and PDAC, 5912 genes, 50.1%(purple) (**C**) Volcano plot of CP, PDAC-CP, fold-change vs P-value for genes (**D**) Splicing index of AKR1B1 (− 9.65) down regulated gene in PDAC-CP tissue, red and green lines represent splicing index between CP and PDAC-CP (**E**) Splicing index of KMT2C (− 59.4) downregulated gene in PDAC-CP tissue, red and green lines represent splicing index between CP and PDAC-CP

Transcriptome datasets were generated in pancreatic tissues with CP, PDAC-CP and PDAC (Fig.4 and Additional file 1: Fig. S2). Comparison of transcriptome data between controls and CP identified 4902 genes to be dysregulated (2622 upregulated and 2280 downregulated). Likewise, comparison of data between controls and PDAC-CP identified a total of 6691 genes to be dysregulated (3571upregulated and 3120 downregulated). While comparison of controls and PDAC identified 7350 genes to be dysregulated (5421 upregulated and 1929 downregulated).

### Integrating genes corresponding to altered metabolites and transcriptome data set

We first compared transcriptome data sets of CP vs control and PDAC-CP vs control. The dysregulated genes in CP vs control (4902 genes), PDAC-CP vs control (6691 genes) were then compared with 517 dysregulated genes obtained from altered metabolites separately. This resulted in identifying a total of 102 genes common between transcriptome and metabolome data sets (Fig. 3B). Distribution of 102 genes and types of metabolites are represented in (Fig. 3C*) (*Additional file 5: Dataset S4). Of the 102 genes, 28 in CP (23 upregulated and 5 downregulated) and 33 genes in PDAC-CP (1 upregulated and 32 downregulated; *AKR1B1*, − 2.76*,* Fig. 4D and *KMT2C,* − 2.67 Fig. 4E were tumour suppressors) were unique. The uniquely upregulated gene in PDAC-CP was SPHK1(Fig. 4A). Of the remaining 41 genes, three (*PIGC*, *PKM* and *PPIB)* were commonly upregulated with oncogenic potential (Fig. 4B) and 31 were commonly downregulated among CP and PDAC-CP (Fig. 4C), of which, 3 (*AZGP1, EGLN1 and GNMT*) were tumour suppressors (Fig. 3A).

### Integrating genes corresponding to altered metabolites with transcriptome data in PDAC with a background of CP and PDAC

Integrating 517 genes corresponding to altered metabolites and comparing with transcriptomes datasets of PDAC-CP and PDAC resulted in a total 127 genes that were dysregulated. There were 11 dysregulated genes common between PDAC-CP and PDAC; of which 3 were upregulated with oncogenic potential (*PIGC*, *PKM* and *PPIB*) and 8 were downregulated with no functional relevance. While 24 genes were down regulated (tumour suppressors *AKR1B1* and *KMT2C*), *SPHK1*(2.11 fold) was the only upregulated gene in PDAC-CP. Splicing index of identified genes are listed in Additional file 6: Dataset S5.

### GO enrichment

We performed gene ontology (GO) enrichment analysis using PANTHER for the 102 dysregulated genes and found that majority of them were with hydrolase and transferase activity with predominant cytoplasmic localization affecting metabolic activity (Additional file 1: Fig. S4).

### SPHK1 expression in pancreatic tissues by immunohistochemistry

**Fig. 5:**
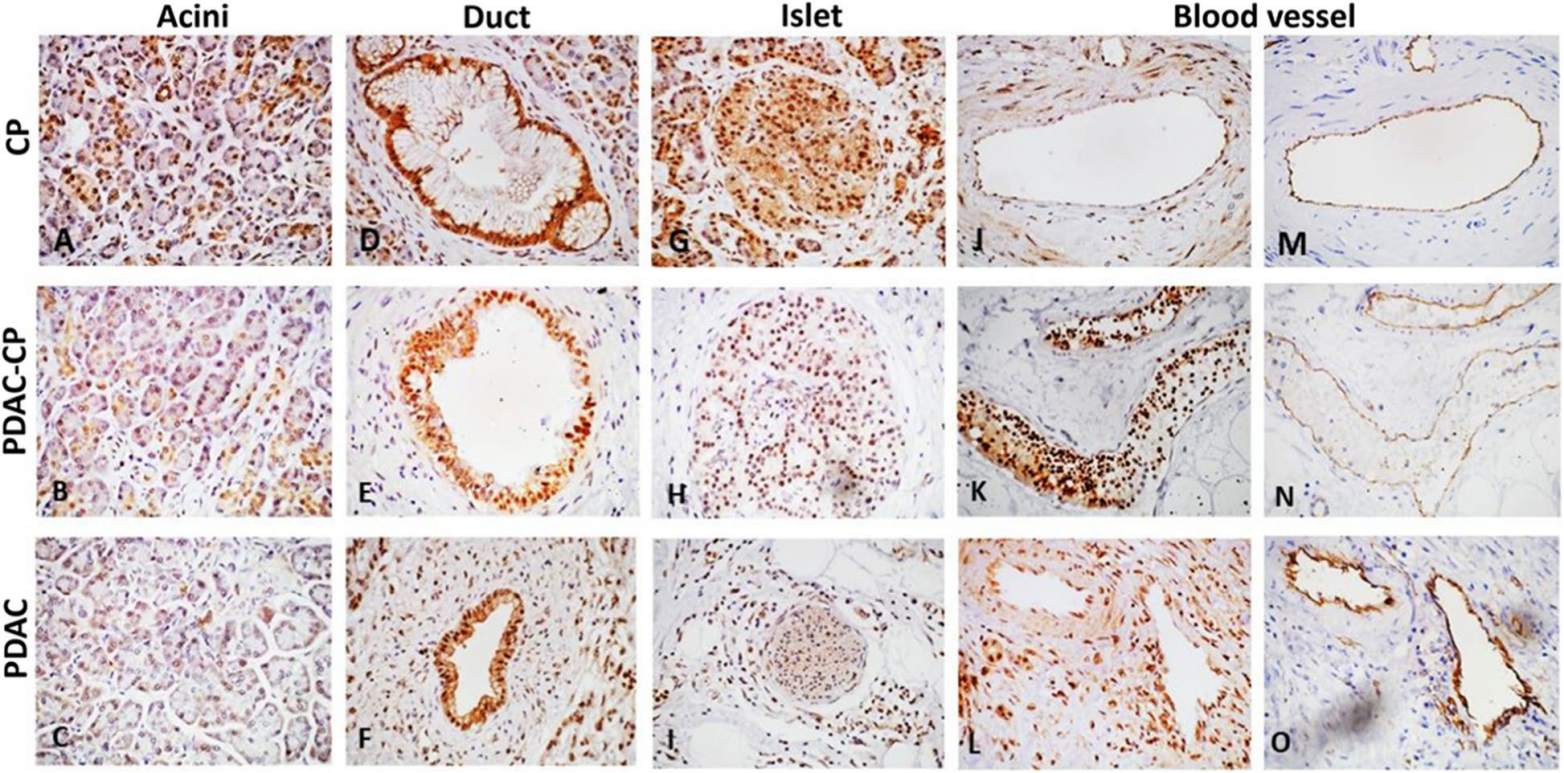
Representative images of SPHK1 expression in acini (**A**)-(**C**), duct (**D**)-(**F**), islet (**G**-**I**), blood vessel(**J**-**L**) and CD31 expression in blood vessel(**M**-**O**) among CP, PDAC-CP and PDAC. Images were captured using Olympus BX63 microscope, 40X objective, scale bar = 10 μm

SPHK1 expression in acini and islets was comparatively low in PDAC-CP and PDAC in comparison to CP (H-score: acini- CP 262, PDAC-CP 138, PDAC 133; Islets-CP 250, PDAC-CP 150, PDAC 163). While the expression was marginally high in ducts (H-score: CP 175, PDAC-CP 193, PDAC 238), SPHK1 expression was significantly high in blood vessels in PDAC-CP (high positives 12/13, low positives 7/15) in comparison to CP (high positives 1/13, low positives 8/15) with a *P-*Value (0.015) (H-score: blood vessels- CP 189, PDAC-CP 242, PDAC 233), Fig. 5, Additional file 1: Table S5.

## Discussion

Earlier studies performed systematic review on metabolomics based diagnostic biomarkers and meta-analysis on cancer risk and clinical trials to determine survival trends in Pancreatic Cancer/PDAC [39–41]. We conducted a systematic review, meta-analysis of studies reporting altered metabolites in CP and PDAC, integrated with human pancreatic transcriptome, and identified enhanced expression of *SPHK1* a known oncogene in PDAC-CP.

Systematic review of metabolic profiles in CP and PDAC identified 25 studies, among them meta-analysis was conducted on 19 studies as they reported AUC, sensitivity and specificity. These studies employed plasma/serum for metabolomics and developed panels comprising of lipids, amino acids, carbohydrates and other organic compounds to differentiate PDAC from CP/healthy controls. There were differences in the panels and biomarkers in different studies probably because of different methodology used and differences in sample size. Therefore, we sub-grouped the metabolites and demonstrated that lipids are superior in differentiating PDAC from CP as compared to amino acids and carbohydrates from the forest plots. Further, pathway enrichment analysis of all metabolites among HC vs PDAC, HC/CP vs PDAC and CP vs PDAC groups employing metaboAnalyst showed glycerophospholipid pathway in HC vs PDAC, glutamate and arginine metabolic pathway in HC/CP vs PDAC and sphingomyelin, TCA intermediates in CP vs PDAC groups involving 30 metabolites. In addition, a comprehensive intermediary metabolic crosstalk generated from these 30 metabolites resulted in 12 different metabolic pathways including phospholipid and sphingomyelin pathways (Fig. 3A).

**Fig. 6:**
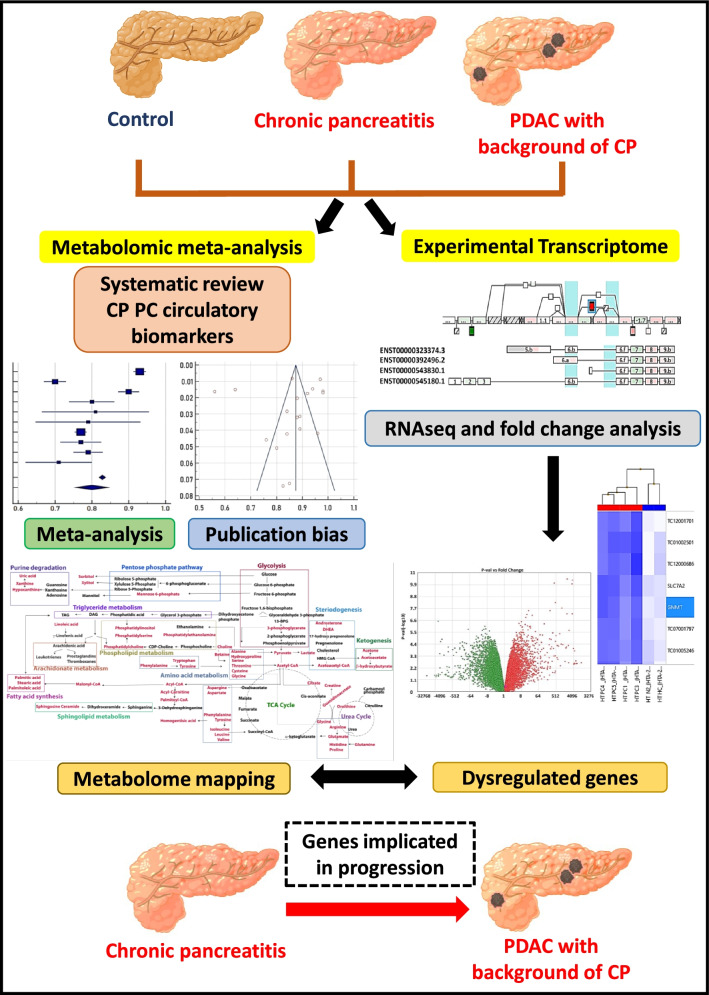
Integration of metabolome data derived from meta-analysis and experimental transcriptome among healthy control, CP, and PDAC-CP patients.

Since our aim was to identify genes of altered metabolic pathways in PDAC-CP, further analysis was focussed on the 30 metabolites in CP and PDAC by retrieving the corresponding genes (*n =* 517) from relational data bases of these circulating metabolites. In order to confirm whether these are the reflection of tissue metabolism, genes data set (derived from circulatory metabolites) were compared with the human transcriptome data set (pancreatic tissues) from our laboratory, Fig. 6. Comparison of gene set from metabolites with gene set from transcriptome yielded 102 genes common between CP and PDAC-CP indicating that these genes may be involved in the transformation of inflammatory lesions to malignant lesions. Some of the most significantly dysregulated genes in our dataset (Additional file 1: Table S3) after integration with transcriptome were enzymes associated with sphingolipid, ceramide and phospholipid metabolism.

Of these 102 genes that were common in CP and PDAC-CP, 3 genes were upregulated; *PIGC*, *PKM*, *PPIB* common to both CP and PDAC-CP (Additional file 1: Fig. S3), with known oncogenic potential. In addition, 3 tumour suppressors genes were down regulated: *GNMT*, *AZGP1* and *EGLN1* (Additional file 1: Fig. S3). Increased expression of tumorigenic genes and decreased expression of tumour suppressor genes suggest a role for these genes in PDAC-CP. Interestingly, *SPHK1*, with known oncogenic characteristics was the only gene upregulated with > 2.0-fold change and tumour suppressors namely *AKR1B1* and *KMT2C* were down regulated in PDAC-CP.

In addition, we also compared transcriptome datasets from PDAC-CP and PDAC with genes corresponding to altered metabolites. Interestingly, among the 127 genes that were dysregulated (Additional file 1: Table S4), the only gene with an upregulation of 2 fold was *SPHK1* seen in the PDAC-CP, but not PDAC. Although, upregulation of *SPHK1* is reported in multiple cancers including PDAC [42–44], our transcriptome data in PDAC was less than the cut off of 2-fold change.

*SPHK1* catalyses generation of Sphingosine-1phosphate (S1P) from ceramide. *SPHK1* is involved in regulation of multiple cellular processes, such as cell survival, cellular migration, angiogenesis and cancer progression. Our meta-analysis/pathway enrichment analysis by metaboAnalyst and integration of corresponding genes of altered metabolite with transcriptome revealed Sphingomyelin pathway and *SPHK1* upregulation pointing to its potential role in early neoplastic transformation of inflammatory lesions in long standing chronic pancreatitis patients. However, the genetic and epigenetic mechanisms of *SPHK1* upregulation need to be further explored. Enrichment of TCA cycle intermediates by metaboAnalyst along with sphingomyelin pathway favours aerobic glycolysis as a requirement for cancer cells [45] when the cancer cells reprogram their metabolic pathways by switching on the aerobic glycolysis, due to their need for mitochondrial ATP, for rapid cell division [46]. Conversion of various metabolites to TCA cycle intermediates is a channel for the cancer cells to fuel their needs and also for the exchange of nutrients [47].

Among the commonly dysregulated genes PIGC showed upregulation in both CP and PDAC-CP indicating that it may have a role. PIGC catalyses biosynthesis of glycosylphosphatidylinositol [48] and its dysregulation was implicated in the pathogenesis of several malignant tumour types [49]. Elevated expression of Phosphatidylinositol glycan anchor biosynthesis class C (PIGC) was shown to promote proliferation and cancer cell migration [50]. However, these findings have to be studied in the context of CP progressing to PDAC under in vivo conditions. Our data also shows down regulation of tumour suppressor genes *AZGP1*, *EGLN1* and *GNMT* of which *AZGP1*, a Zinc α2-glycoprotein which when silenced was shown to increase invasiveness of pancreatic cancer by induction of mesenchymal transition [51]. Our analysis showed 10-fold decrease in *AZGP1* in PDAC-CP, indicating its importance. *AKR1B1* has been implicated in physiological and biochemical pathways, such as carbohydrate metabolism, inflammation and prostaglandin synthesis [52, 53]. Our data also showed down regulation of *KMT2C*, which is known to regulate DNA repair [54, 55]. It’s down regulation concomitant with dysregulation of oncogenes and tumour suppressors may lead to malignant transformation of inflammatory lesions.

Our results demonstrate that SPHK1 was differentially expressed in acini, islets, ducts and blood vessels in CP, PDAC-CP and PDAC. SPHK1/S1P signalling is known to play a crucial role in inflammation, cell migration, and vascular development. Sphk1 is widely upregulated across a diverse range of human cancers and has been inextricably linked to tumorigenesis [56–60]. *SPHK1* was earlier reported to be upregulated in pancreatic ductal adenocarcinoma demonstrating resistance to gemcitabine [61], its regulation of angiogenesis [62] and miRNA 506/SPHK1 axis as a therapeutic target [63]. Upregulation of SPHK1 expression in the current study shows a novel role of SPHK1/S1P axis during the progression of CP to PDAC-CP angiogenesis. The exact mechanism of angiogenic factor secretion during the progression of CP to PDAC-CP and pancreas cell type S1PRs (Sphingosine 1 phosphate receptors) needs to be further characterized.

Limitation of the study is small size of the tissue transcriptomes and validation sets. We have also performed RNA sequencing on NGS (Ion Proton; Life technologies) to confirm the dysregulated genes. Despite small sample size, validation of SPHK1 in pancreatic tissues confirms higher expression in blood vessels indicating that SPHK1 has a role in angiogenesis during progression of CP to PDAC-CP. Data from both the platforms were comparable and therefore our results are reliable and may be suitable candidates for further validation in a large number of samples before it can be used in clinical practice.

In conclusion, the identification of sphingomyelin pathway from metaboAnalyst, upregulation of *SPHK1* in the integration of genes associated with metabolome and transcriptome (CP and PDAC-CP) and comparison of transcriptome data between PDAC-CP and PDAC, both indicate a role for *SPHK1*in the malignant transformation of inflammatory lesions in CP. *SPHK1* needs to be studied further for exploiting its diagnostic and therapeutic potential in long standing CP patients.

## Supplementary Information

**Additional file 1: Fig. S1.** Publication bias and Sensitivity plots (*A)* Funnel plot of metabolomic studies (*B)* Meta-analysis based on reported sensitivity among 10 studies. Heterogenity (I^2^) was assessed for fixed and random effects (*C)* Publication bias computed from AUC and calculated standard error among 19 studies. **Fig. S2.** Representative heat map of transcriptome datasets *(A)* Significant (P-value ≤ 0.05) dysregulated genes in tissues with Chronic pancreatitis compared to Controls sorted *B)* Significant (P-value ≤ 0.05) dysregulated genes in tissues with PDAC with a background of chronic pancreatitis compared to Controls, *(C)* Significant (P-value ≤ 0.05) dysregulated genes in tissues with Pancreatic ductal adenocarcinoma compared to Controls. Red and green colour depict up and downregulated genes. **Fig. S3.** Transcriptomics (*A*) Splicing index of 3 upregulated genes *PIGC*(21.63), *PKM*(-4.92), *PPIB*(3.51) in CP and PDAC-CP tissues. Red and green lines represent splicing index between CP and PDAC-CP for each gene respectively (*B*) Heatmap of 3 tumor suppressor genes *AZGP1*, *EGLN1* and *GNMT* in CP and PDAC-CP tissues. Fig. S4. Gene ontology of 102 genes that were common between CP and PDAC-CP (*A)* Molecular function of corresponding genes of altered metabolites identified in CP and PDAC and transcriptomes of CP, PDAC-CP (*B)* Biological processes associated with genes identified in CP, PDAC metabolome and CP, PDAC-CP transcriptomes (*C)* Cellular component distribution for genes identified in CP, PC metabolome and CP, PDAC-CP transcriptomes (*D)* Protein class distribution for genes identified in CP, PC metabolomics and CP, PDAC-CP transcriptomes (*E)* Pathway analysis for genes identified in CP, PC metabolomes and CP, PDAC-CP transcriptomes. **Table S1.** MOOSE guidelines. **Table S2.** Characteristics of patients for pancreatic tissue transcriptomes. **Table S3.** Dysregulated genes in Chronic pancreatitis(CP) and Pancreatic ductal adenocarcinoma with a background of chronic pancreatitis(PDAC-CP), tumor suppressor genes are highlighted in yellow color. **Table S4.** Dysregulated genes in Pancreatic ductal adenocarcinoma with a background of chronic pancreatitis(PDAC-CP) and PDAC, tumor suppressor genes are highlighted in yellow color. **Table S5.** Fisher’s exact test between CP, PDAC-CP and PC groups.

**Additional file 2: Dataset S1.** Circulatory metabolites identified in chronic pancreatitis, pancreatic cancer and pancreatic ductal adenocarcinoma using metabolomics platforms.

**Additional file 3: Dataset S2.** Publication bias funnel plot, sensitivity forest plot among the studies reporting circulatory metabolites.

**Additional file 4: Dataset S3.** Metabolites list derived from metabolomics studies comparing healthy controls and pancreatic cancer/PDAC; healthy controls/chronic pancreatitis and pancreatic cancer/PDAC; chronic pancreatitis and pancreatic cancer/PDAC.

**Additional file 5: Dataset S4.** List of genes derived from relational databases.

**Additional file 6: Dataset S5.** Splicing index of dysregulated genes.

## Data Availability

Transcriptome datasets are available online: Vishnubhotla, Ravikanth (2021), “Transcriptome Dataset”, Mendeley Data, V1, doi: 10.17632/2jjbpvfm72.1

## Abbreviations

CP: Chronic pancreatitis
PDAC-CP: Pancreatic ductal adenocarcinoma with a background of CP
PDAC: Pancreatic ductal adenocarcinom

## References

[1] Collaborators GBDPC (2019). The global, regional, and national burden of pancreatic cancer and its attributable risk factors in 195 countries and territories, 1990-2017: a systematic analysis for the global burden of disease study 2017. Lancet. Gastroenterol Hepatol.

[2] Munigala S, Singh A, Gelrud A, Agarwal B (2015). Predictors for pancreatic Cancer diagnosis following new-onset diabetes mellitus. Clin Transl Gastroenterol.

[3] Brodovicz KG, Kou TD, Alexander CM, O'Neill EA, Engel SS, Girman CJ (2012). Impact of diabetes duration and chronic pancreatitis on the association between type 2 diabetes and pancreatic cancer risk. Diabetes Obes Metab.

[4] Siegel RL, Miller KD, Jemal A (2018). Cancer statistics, 2018. CA Cancer J Clin.

[5] Hezel AF, Kimmelman AC, Stanger BZ, Bardeesy N, Depinho RA (2006). Genetics and biology of pancreatic ductal adenocarcinoma. Genes Dev.

[6] Makohon-Moore A, Iacobuzio-Donahue CA (2016). Pancreatic cancer biology and genetics from an evolutionary perspective. Nat Rev Cancer.

[7] Spratlin JL, Serkova NJ, Eckhardt SG (2009). Clinical applications of metabolomics in oncology: a review. Clin Cancer Res.

[8] Stroup DF, Berlin JA, Morton SC, Olkin I, Williamson GD, Rennie D (2000). Meta-analysis of observational studies in epidemiology: a proposal for reporting. Meta-analysis of observational studies in epidemiology (MOOSE) group. JAMA..

[9] Io M, Eden J, Levit L, Berg A, Morton S (2011). Finding what works. Health care: standards for systematic reviews.

[10] Sumner LW, Amberg A, Barrett D, Beale MH, Beger R, Daykin CA (2007). Proposed minimum reporting standards for chemical analysis chemical analysis working group (CAWG) metabolomics standards initiative (MSI). Metabolomics..

[11] Bossuyt PM, Reitsma JB, Bruns DE, Gatsonis CA, Glasziou PP, Irwig LM (2003). Towards complete and accurate reporting of studies of diagnostic accuracy: the STARD initiative. AJR Am J Roentgenol.

[12] Turakhia MP, Sabatine MS (2017). How we evaluate biomarker studies. JAMA Cardiol.

[13] Schoonjans F, Zalata A, Depuydt CE, Comhaire FH (1995). MedCalc: a new computer program for medical statistics. Comput Methods Prog Biomed.

[14] Wishart DS, Feunang YD, Marcu A, Guo AC, Liang K, Vazquez-Fresno R (2018). HMDB 4.0: the human metabolome database for 2018. Nucleic Acids Res.

[15] Xia J, Wishart DS (2016). Using MetaboAnalyst 3.0 for Comprehensive Metabolomics Data Analysis. Curr Protoc Bioinformatics.

[16] Kanehisa M, Furumichi M, Sato Y, Ishiguro-Watanabe M, Tanabe M (2021). KEGG: integrating viruses and cellular organisms. Nucleic Acids Res.

[17] Pang Z, Chong J, Zhou G, de Lima Morais DA, Chang L, Barrette M (2021). MetaboAnalyst 5.0: narrowing the gap between raw spectra and functional insights. Nucleic Acids Res.

[18] Mi H, Muruganujan A, Huang X, Ebert D, Mills C, Guo X (2019). Protocol update for large-scale genome and gene function analysis with the PANTHER classification system (v.14.0). Nat Protoc.

[19] Topaz N, Mojib N, Chande AT, Kubanek J, Jordan IK. RampDB: a web application and database for the exploration and prediction of receptor activity modifying protein interactions. Database (Oxford). 2017;2017. 10.1093/database/bax067 PMC5737055 29220456

[20] Good BM, Van Auken K, Hill DP, Mi H, Carbon S, Balhoff JP, et al. Reactome and the gene ontology: digital convergence of data resources. Bioinformatics. 2021. 10.1093/bioinformatics/btab325 PMC8504636 33964129

[21] Zhao M, Kim P, Mitra R, Zhao J, Zhao Z (2016). TSGene 2.0: an updated literature-based knowledgebase for tumor suppressor genes. Nucleic Acids Res.

[22] Varghese F, Bukhari AB, Malhotra R, De A (2014). IHC profiler: an open source plugin for the quantitative evaluation and automated scoring of immunohistochemistry images of human tissue samples. PLoS One.

[23] McClelland RA, Finlay P, Walker KJ, Nicholson D, Robertson JF, Blamey RW (1990). Automated quantitation of immunocytochemically localized estrogen receptors in human breast cancer. Cancer Res.

[24] Fukutake N, Ueno M, Hiraoka N, Shimada K, Shiraishi K, Saruki N (2015). A novel multivariate index for pancreatic Cancer detection based on the plasma free amino acid profile. PLoS One.

[25] Hirata Y, Kobayashi T, Nishiumi S, Yamanaka K, Nakagawa T, Fujigaki S (2017). Identification of highly sensitive biomarkers that can aid the early detection of pancreatic cancer using GC/MS/MS-based targeted metabolomics. Clin Chim Acta.

[26] Xie G, Lu L, Qiu Y, Ni Q, Zhang W, Gao YT (2015). Plasma metabolite biomarkers for the detection of pancreatic cancer. J Proteome Res.

[27] Suzuki M, Nishiumi S, Kobayashi T, Sakai A, Iwata Y, Uchikata T (2017). Use of on-line supercritical fluid extraction-supercritical fluid chromatography/tandem mass spectrometry to analyze disease biomarkers in dried serum spots compared with serum analysis using liquid chromatography/tandem mass spectrometry. Rapid Commun Mass Spectrom.

[28] Urayama S, Zou W, Brooks K, Tolstikov V (2010). Comprehensive mass spectrometry based metabolic profiling of blood plasma reveals potent discriminatory classifiers of pancreatic cancer. Rapid Commun Mass Spectrom.

[29] Zhang X, Shi X, Lu X, Li Y, Zhan C, Akhtar ML (2020). Novel metabolomics serum biomarkers for pancreatic ductal adenocarcinoma by the comparison of pre-, Postoperative and Normal Samples. J Cancer.

[30] Mayerle J, Kalthoff H, Reszka R, Kamlage B, Peter E, Schniewind B (2018). Metabolic biomarker signature to differentiate pancreatic ductal adenocarcinoma from chronic pancreatitis. Gut..

[31] Fest J, Vijfhuizen LS, Goeman JJ, Veth O, Joensuu A, Perola M (2019). Search for early pancreatic Cancer blood biomarkers in five European prospective population biobanks using metabolomics. Endocrinology..

[32] Mayers JR, Wu C, Clish CB, Kraft P, Torrence ME, Fiske BP (2014). Elevation of circulating branched-chain amino acids is an early event in human pancreatic adenocarcinoma development. Nat Med.

[33] Leichtle AB, Ceglarek U, Weinert P, Nakas CT, Nuoffer JM, Kase J (2013). Pancreatic carcinoma, pancreatitis, and healthy controls: metabolite models in a three-class diagnostic dilemma. Metabolomics..

[34] Di Gangi IM, Mazza T, Fontana A, Copetti M, Fusilli C, Ippolito A (2016). Metabolomic profile in pancreatic cancer patients: a consensus-based approach to identify highly discriminating metabolites. Oncotarget..

[35] Elebo N, Omoshoro-Jones J, Fru PN, Devar J, De Wet van Zyl C, Vorster BC, et al. Serum Metabolomic and lipoprotein profiling of pancreatic ductal adenocarcinoma patients of African ancestry. Metabolites. 2021;11(10). 10.3390/metabo11100663 PMC8540259 34677378

[36] Zhang L, Jin H, Guo X, Yang Z, Zhao L, Tang S (2012). Distinguishing pancreatic cancer from chronic pancreatitis and healthy individuals by (1) H nuclear magnetic resonance-based metabonomic profiles. Clin Biochem.

[37] Bathe OF, Shaykhutdinov R, Kopciuk K, Weljie AM, McKay A, Sutherland FR (2011). Feasibility of identifying pancreatic cancer based on serum metabolomics. Cancer Epidemiol Biomark Prev.

[38] Beger RD, Schnackenberg LK, Holland RD, Li D, Dragan Y (2006). Metabonomic models of human pancreatic cancer using 1D proton NMR spectra of lipids in plasma. Metabolomics..

[39] Kirkegard J, Mortensen FV, Cronin-Fenton D (2017). Chronic pancreatitis and pancreatic Cancer risk: a systematic review and Meta-analysis. Am J Gastroenterol.

[40] Hall BR, Cannon A, Atri P, Wichman CS, Smith LM, Ganti AK (2018). Advanced pancreatic cancer: a meta-analysis of clinical trials over thirty years. Oncotarget..

[41] Long NP, Yoon SJ, Anh NH, Nghi TD, Lim DK, Hong YJ (2018). A systematic review on metabolomics-based diagnostic biomarker discovery and validation in pancreatic cancer. Metabolomics..

[42] Aoki H, Aoki M, Katsuta E, Ramanathan R, Idowu MO, Spiegel S (2016). Host sphingosine kinase 1 worsens pancreatic cancer peritoneal carcinomatosis. J Surg Res.

[43] Long J, YaoYi Sui Z, Sui Y, Fang S. SphK1 promotes Cancer progression through activating JAK/STAT pathway and U*p-*regulating S1PR1 expression in Colon Cancer cells. Anti Cancer Agents Med Chem. 2021. 10.2174/1871520621666210401105344 33797381

[44] Long J, Xie Y, Yin J, Lu W, Fang S (2016). SphK1 promotes tumor cell migration and invasion in colorectal cancer. Tumour Biol.

[45] Anderson NM, Mucka P, Kern JG, Feng H (2018). The emerging role and targetability of the TCA cycle in cancer metabolism. Protein Cell.

[46] Blum R, Kloog Y (2014). Metabolism addiction in pancreatic cancer. Cell Death Dis.

[47] Hui S, Ghergurovich JM, Morscher RJ, Jang C, Teng X, Lu W (2017). Glucose feeds the TCA cycle via circulating lactate. Nature..

[48] Watanabe R, Inoue N, Westfall B, Taron CH, Orlean P, Takeda J (1998). The first step of glycosylphosphatidylinositol biosynthesis is mediated by a complex of PIG-A, PIG-H, PIG-C and GPI1. EMBO J.

[49] Peng X, Lei C, He A, Luo R, Cai Y, Dong W (2021). Upregulation of phosphatidylinositol glycan anchor biosynthesis class C is associated with unfavorable survival prognosis in patients with hepatocellular carcinoma. Oncol Lett.

[50] Yang L, Gao Z, Hu L, Wu G, Yang X, Zhang L (2016). Glycosylphosphatidylinositol anchor modification machinery deficiency is responsible for the formation of pro-prion protein (PrP) in BxPC-3 protein and increases Cancer cell motility. J Biol Chem.

[51] Kong B, Michalski CW, Hong X, Valkovskaya N, Rieder S, Abiatari I (2010). AZGP1 is a tumor suppressor in pancreatic cancer inducing mesenchymal-to-epithelial transdifferentiation by inhibiting TGF-beta-mediated ERK signaling. Oncogene..

[52] Xiao MB, Jin DD, Jiao YJ, Ni WK, Liu JX, Qu LS (2018). beta2-AR regulates the expression of AKR1B1 in human pancreatic cancer cells and promotes their proliferation via the ERK1/2 pathway. Mol Biol Rep.

[53] Banerjee S. Aldo keto reductases AKR1B1 and AKR1B10 in Cancer: molecular mechanisms and signaling networks. Adv Exp Med Biol. 2021. 10.1007/5584_2021_634 33945128

[54] Rampias T, Karagiannis D, Avgeris M, Polyzos A, Kokkalis A, Kanaki Z, et al. The lysine-specific methyltransferase KMT2C/MLL3 regulates DNA repair components in cancer. EMBO Rep. 2019;20(3). 10.15252/embr.201846821 PMC6399616 30665945

[55] Liu X, Qiu R, Xu M, Meng M, Zhao S, Ji J (2021). KMT2C is a potential biomarker of prognosis and chemotherapy sensitivity in breast cancer. Breast Cancer Res Treat.

[56] Zhu L, Wang Z, Lin Y, Chen Z, Liu H, Chen Y (2015). Sphingosine kinase 1 enhances the invasion and migration of non-small cell lung cancer cells via the AKT pathway. Oncol Rep.

[57] Beach JA, Aspuria PJ, Cheon DJ, Lawrenson K, Agadjanian H, Walsh CS (2016). Sphingosine kinase 1 is required for TGF-beta mediated fibroblastto- myofibroblast differentiation in ovarian cancer. Oncotarget..

[58] Salama MF, Carroll B, Adada M, Pulkoski-Gross M, Hannun YA, Obeid LM (2015). A novel role of sphingosine kinase-1 in the invasion and angiogenesis of VHL mutant clear cell renal cell carcinoma. FASEB journal : official publication of the Federation of American Societies for Experimental Biology.

[59] Kawahara S, Otsuji Y, Nakamura M, Murakami M, Murate T, Matsunaga T (2013). Sphingosine kinase 1 plays a role in the upregulation of CD44 expression through extracellular signal-regulated kinase signaling in human colon cancer cells. Anti-Cancer Drugs.

[60] Bao Y, Guo Y, Zhang C, Fan F, Yang W. Sphingosine Kinase 1 and Sphingosine-1-Phosphate Signaling in Colorectal Cancer. Int J Mol Sci. 2017;18(10). 10.3390/ijms18102109 PMC5666791 28991193

[61] Guillermet-Guibert J, Davenne L, Pchejetski D, Saint-Laurent N, Brizuela L, Guilbeau-Frugier C (2009). Targeting the sphingolipid metabolism to defeat pancreatic cancer cell resistance to the chemotherapeutic gemcitabine drug. Mol Cancer Ther.

[62] Yu M, Zhang K, Wang S, Xue L, Chen Z, Feng N (2021). Increased SPHK1 and HAS2 expressions correlate to poor prognosis in pancreatic Cancer. Biomed Res Int.

[63] Li J, Wu H, Li W, Yin L, Guo S, Xu X (2016). Downregulated miR-506 expression facilitates pancreatic cancer progression and chemoresistance via SPHK1/Akt/NF-kappaB signaling. Oncogene..

