# Correction: Integration of metabolites from meta-analysis with transcriptome reveals enhanced *SPHK1* in PDAC with a background of pancreatitis

**DOI:** 10.1186/s12885-022-09920-7

**Published:** 2022-07-28

**Authors:** Vijayasarathy Ketavarapu, Vishnubhotla Ravikanth, Mitnala Sasikala, G. V. Rao, Ch. Venkataramana Devi, Prabhakar Sripadi, Murali Satyanarayana Bethu, Ramars Amanchy, H. V. V. Murthy, Stephen J. Pandol, D. Nageshwar Reddy

**Affiliations:** 1 Asian Healthcare Foundation, Asian Institute of Gastroenterology, Mindspace Rd, Gachibowli, Hyderabad, Telangana 500032 India; 2 AIG Hospitals, Mindspace Rd, Gachibowli, Hyderabad, Telangana 500032 India; 3 Department of Biochemistry, University College of Science, Osmania University, Hyderabad, 500 007 India; 4 Centre for Mass Spectrometry, Analytical & Structural Chemistry Department, CSIR-Indian Institute of Chemical Technology, Tarnaka, Hyderabad, 500 007 India; 5 Division of Applied Biology, CSIR-IICT (Indian Institute of Chemical Technology), Ministry of Science and Technology (GOI), Hyderabad, Telangana 500007 India; 6 Department of Pharmacology and Therapeutics, Roswell Park Comprehensive Cancer Center, Elm &Carlton Streets, Bufalo, New York, 14221 USA; 7 Department of Medicine, Division of Digestive and Liver Diseases, Cedars-Sinai Medical Center, Los Angeles, CA USA

**Correction: BMC Cancer 22, 792 (2022)**

**https://doi.org/10.1186/s12885-022-09816-6**

Following publication of the original article [1], the authors identified a typesetting error. Figure 2 was published in incomplete form. Figure 2E-G were erroneously omitted. The complete Fig. 2 has been published in this correction. The original article [1] has been updated.

**Fig. 2:**
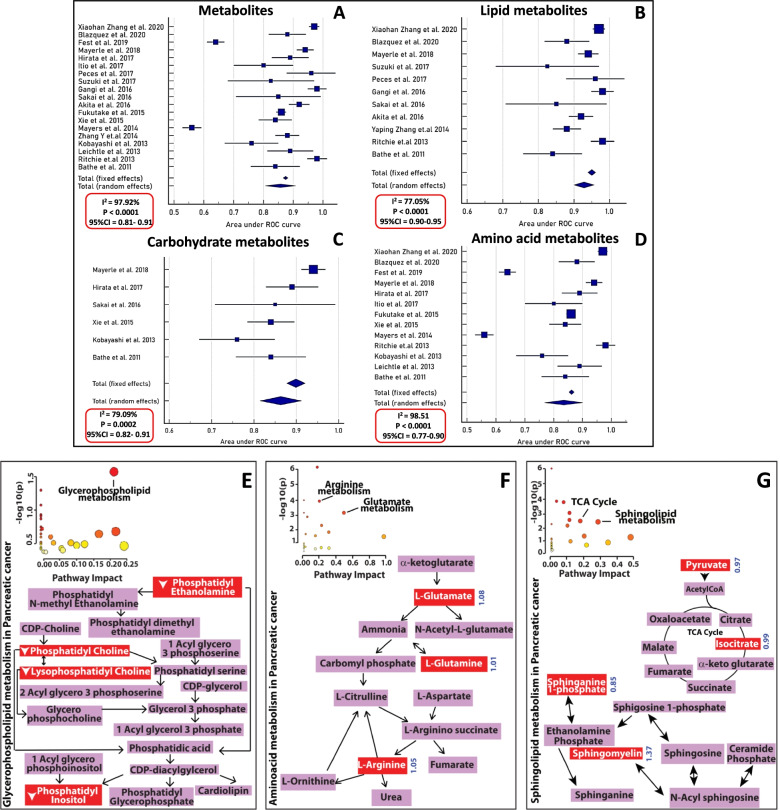
Forest plots of altered metabolites identified in meta-analysis (**A**) Forest plot of 19 metabolomic studies using AUC values and computed standard error. Heterogeneity (I^2^) was assessed for fixed and random effects (**B**) Forest plot of lipid metabolites retrieved from 11 studies (**C**) Forest plot of carbohydrate metabolites retrieved from 6 studies (**D**) Forest plot of amino acid metabolites retrieved from 13 studies. Metabolic pathway analysis using metaboAnalyst identified (**E**) Enriched glycerophospholipid pathway for circulatory metabolites detected in healthy control and PDAC. Metabolites marked in red are altered in PDAC patients as compared to healthy controls (**F**) metaboAnalyst analysis identified enriched arginine and glutamate metabolism for circulatory metabolites detected in PDAC as compared to healthy controls/chronic pancreatitis. Metabolites are marked in red and their fold changes are in blue color (**G**) metaboAnalyst analysis identified enriched sphingomyelin pathway and TCA cycle for circulatory metabolites detected in PDAC and CP patients. Metabolites are marked in red and their fold changes are in blue color
