# Supplementary material for: Integration of metabolites from meta-analysis with transcriptome reveals enhanced SPHK1 in PDAC with a background of pancreatitis
Source: BMC Cancer. 2022 Jul 19;22:792. doi: 10.1186/s12885-022-09816-6 (PMC9295503; doi:10.1186/s12885-022-09816-6)
Supplement: Supplementary file 1 — Additional file 1: Fig. S1. Publication bias and Sensitivity plots (A) Funnel plot of metabolomic studies (B) Meta-analysis based on reported sensitivity among 10 studies. Heterogenity (I2) was assessed for fixed and random effects (C) Publication bias computed from AUC and calculated standard error among 19 studies. Fig. S2. Representative heat map of transcriptome datasets (A) Significant (P-value ≤ 0.05) dysregulated genes in tissues with Chronic pancreatitis compared to Controls sorted B) Significant (P-value ≤ 0.05) dysregulated genes in tissues with PDAC with a background of chronic pancreatitis compared to Controls, (C) Significant (P-value ≤ 0.05) dysregulated genes in tissues with Pancreatic ductal adenocarcinoma compared to Controls. Red and green colour depict up and downregulated genes. Fig. S3. Transcriptomics (A) Splicing index of 3 upregulated genes PIGC(21.63), PKM(-4.92), PPIB(3.51) in CP and PDAC-CP tissues. Red and green lines represent splicing index between CP and PDAC-CP for each gene respectively (B) Heatmap of 3 tumor suppressor genes AZGP1, EGLN1 and GNMT in CP and PDAC-CP tissues. Fig. S4. Gene ontology of 102 genes that were common between CP and PDAC-CP (A) Molecular function of corresponding genes of altered metabolites identified in CP and PDAC and transcriptomes of CP, PDAC-CP (B) Biological processes associated with genes identified in CP, PDAC metabolome and CP, PDAC-CP transcriptomes (C) Cellular component distribution for genes identified in CP, PC metabolome and CP, PDAC-CP transcriptomes (D) Protein class distribution for genes identified in CP, PC metabolomics and CP, PDAC-CP transcriptomes (E) Pathway analysis for genes identified in CP, PC metabolomes and CP, PDAC-CP transcriptomes. Table S1. MOOSE guidelines. Table S2. Characteristics of patients for pancreatic tissue transcriptomes. Table S3. Dysregulated genes in Chronic pancreatitis(CP) and Pancreatic ductal adenocarcinoma with a background of chronic pancreatitis(PDAC-CP) [file 12885_2022_9816_MOESM1_ESM.docx]

**Supplementary Information for**

**Integration of metabolites from meta-analysis with transcriptome reveals enhanced *SPHK1* in PDAC with a background of pancreatitis**

Vijayasarathy Ketavarapu^1^, Vishnubhotla Ravikanth^1^, Mitnala Sasikala^1^, G.V. Rao^2^, Ch. Venkata Ramana Devi^3^, Prabhakar Sripadi^4^, Murali Satyanarayana Bethu^5,6^, Ramars Amanchy^5^, H.V.V. Murthy^1^, Stephen J Pandol^7^, D. Nageshwar Reddy^3^

**Author Affiliations**:

^1^Asian Healthcare Foundation, Asian Institute of Gastroenterology, Mindspace Rd, Gachibowli, Hyderabad, Telangana, India 500032

^2^ AIG Hospitals, Mindspace Rd, Gachibowli, Hyderabad, Telangana, India 500032

^3^Department of Biochemistry, University College of Science, Osmania University, Hyderabad 500 007, India

^4^Centre for Mass Spectrometry, Analytical & Structural Chemistry Department, CSIR-Indian Institute of Chemical Technology, Tarnaka, Hyderabad 500 007, India

^5^Division of Applied Biology, CSIR-IICT (Indian Institute of Chemical Technology), Ministry of Science and Technology (GOI), Hyderabad 500007, Telangana, India

^6^Department of Pharmacology and Therapeutics, Roswell Park Comprehensive Cancer Center, Elm &Carlton Streets, Buffalo, New York, 14221, USA

^7^Department of Medicine, Division of Digestive and Liver Diseases, Cedars-Sinai Medical Center, Los Angeles, CA, USA.

Sasikala Mitnala

Email: drsasikala.mitnala@aighospitals.com

**This supplementary information file includes:**

Figures S1 to S4

Tables S1 to S5

Legends for Datasets S1 to S5

**Other supplementary materials for this manuscript include the following:**

Datasets S1 to S5


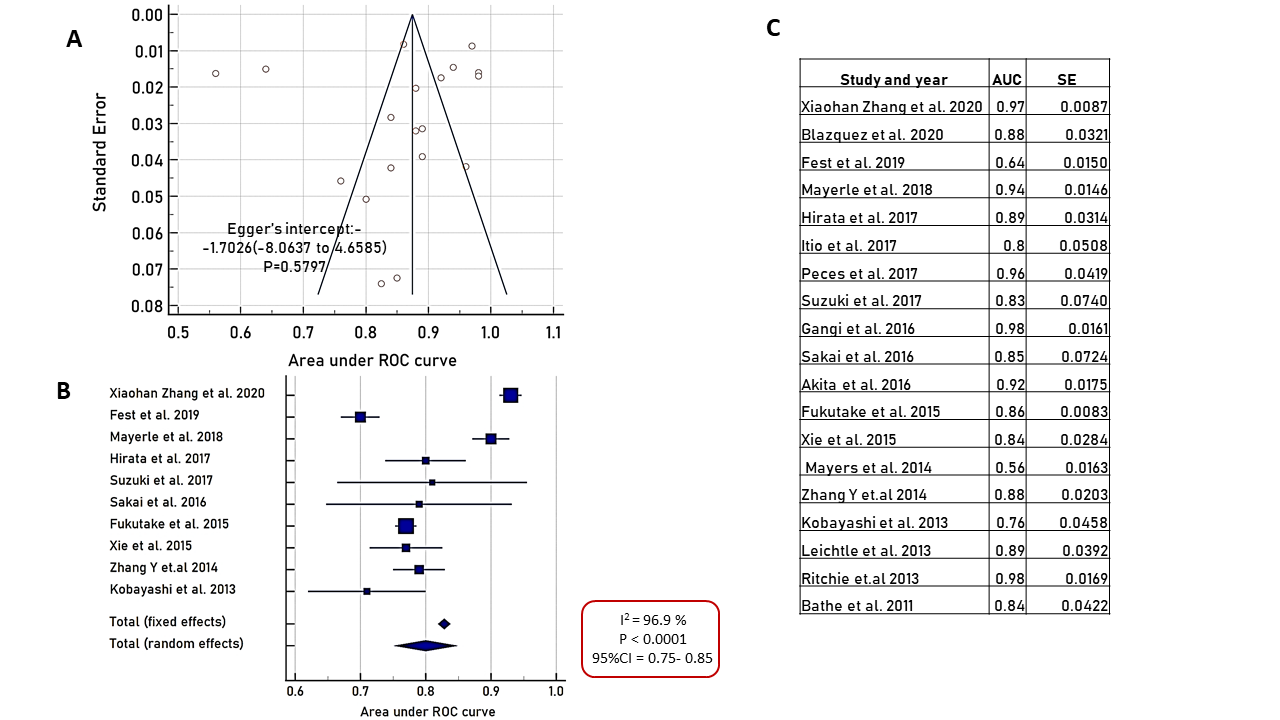


**Fig. S1**. Publication bias and Sensitivity plots (*A)* Funnel plot of metabolomic studies (*B)* Meta-analysis based on reported sensitivity among 10 studies. Heterogenity (I^2^) was assessed for fixed and random effects (*C)* Publication bias computed from AUC and calculated standard error among 19 studies

**
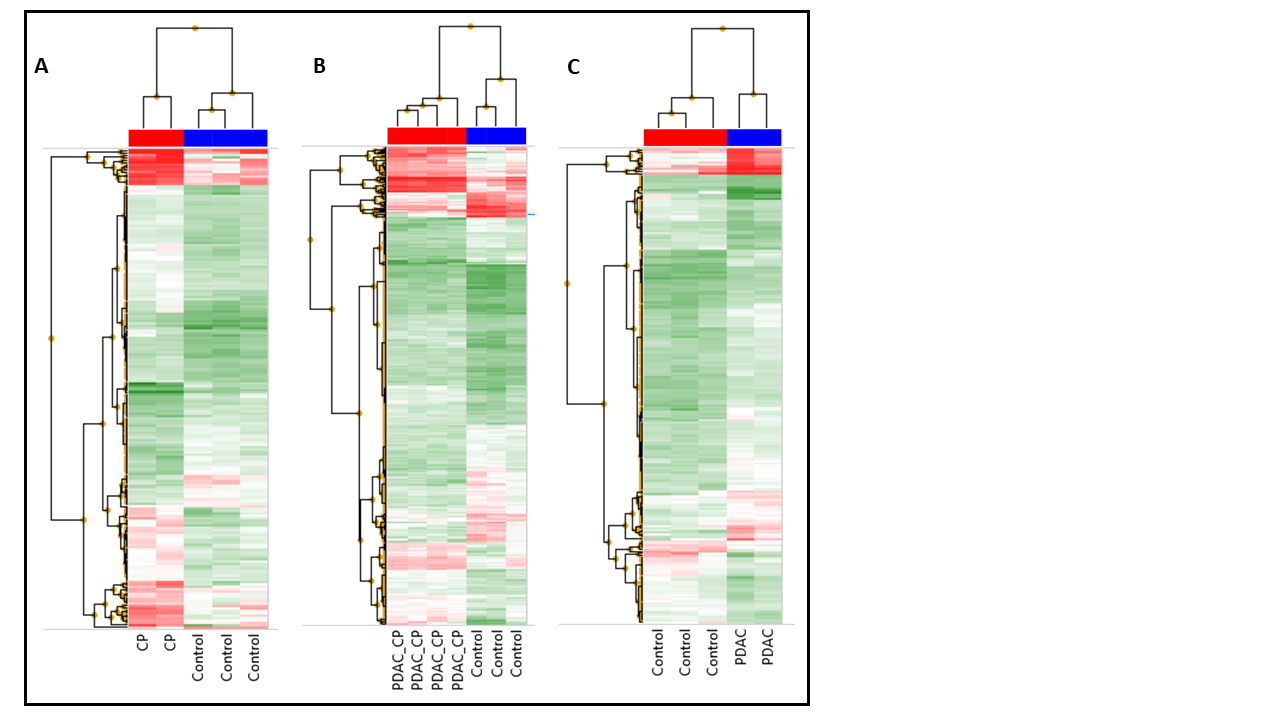
**

**Fig. S2.** Representative heat map of transcriptome datasets *(A)* Significant (P-value ≤ 0.05) dysregulated genes in tissues with Chronic pancreatitis compared to Controls sorted *B)* Significant (P-value ≤ 0.05) dysregulated genes in tissues with PDAC with a background of chronic pancreatitis compared to Controls, *(C)* Significant (P-value ≤ 0.05) dysregulated genes in tissues with Pancreatic ductal adenocarcinoma compared to Controls. Red and green colour depict up and downregulated genes.

**
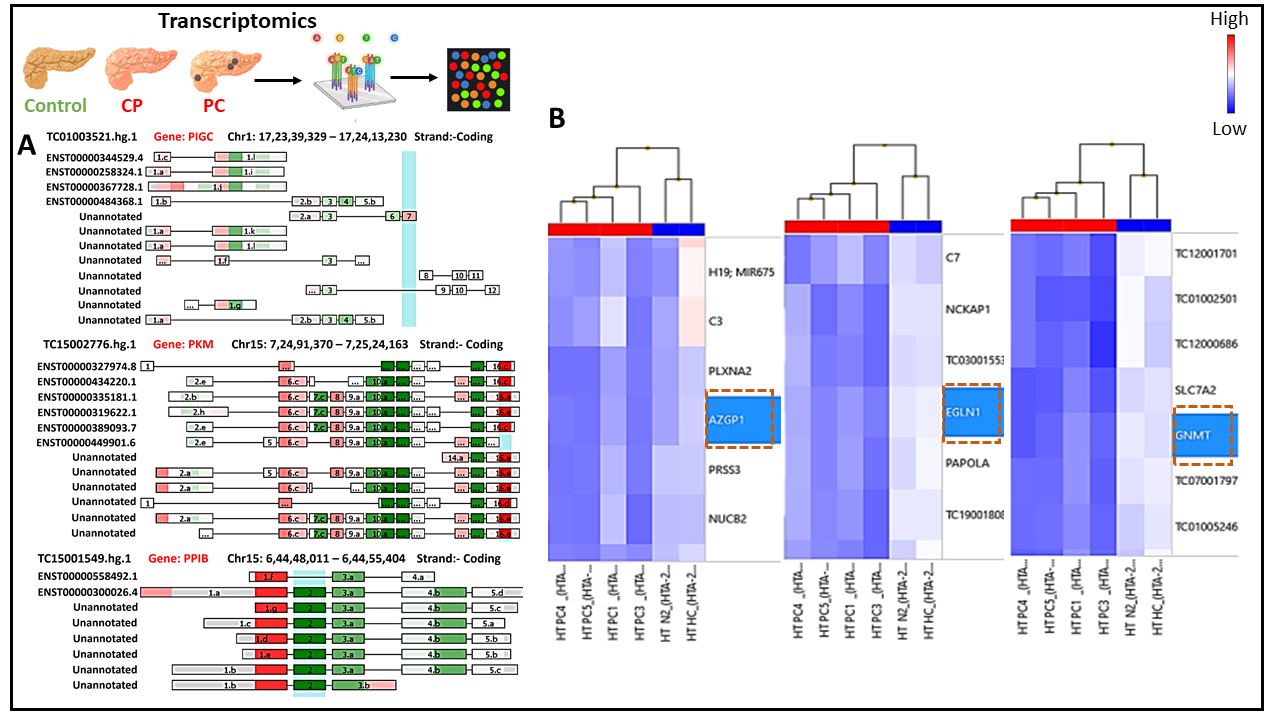
**

**Fig. S3.** Transcriptomics (*A*) Splicing index of 3 upregulated genes *PIGC*(21.63), *PKM*(-4.92), *PPIB*(3.51) in CP and PDAC-CP tissues. Red and green lines represent splicing index between CP and PDAC-CP for each gene respectively (*B*) Heatmap of 3 tumor suppressor genes *AZGP1*, *EGLN1* and *GNMT* in CP and PDAC-CP tissues


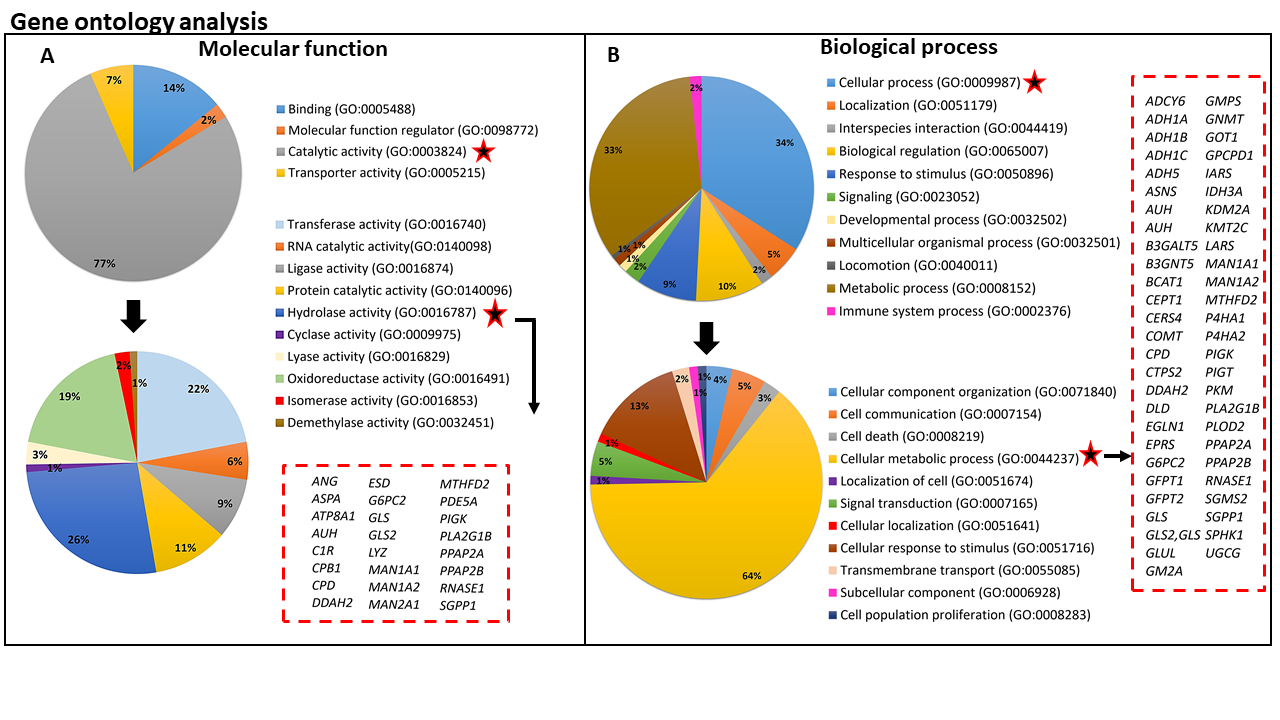

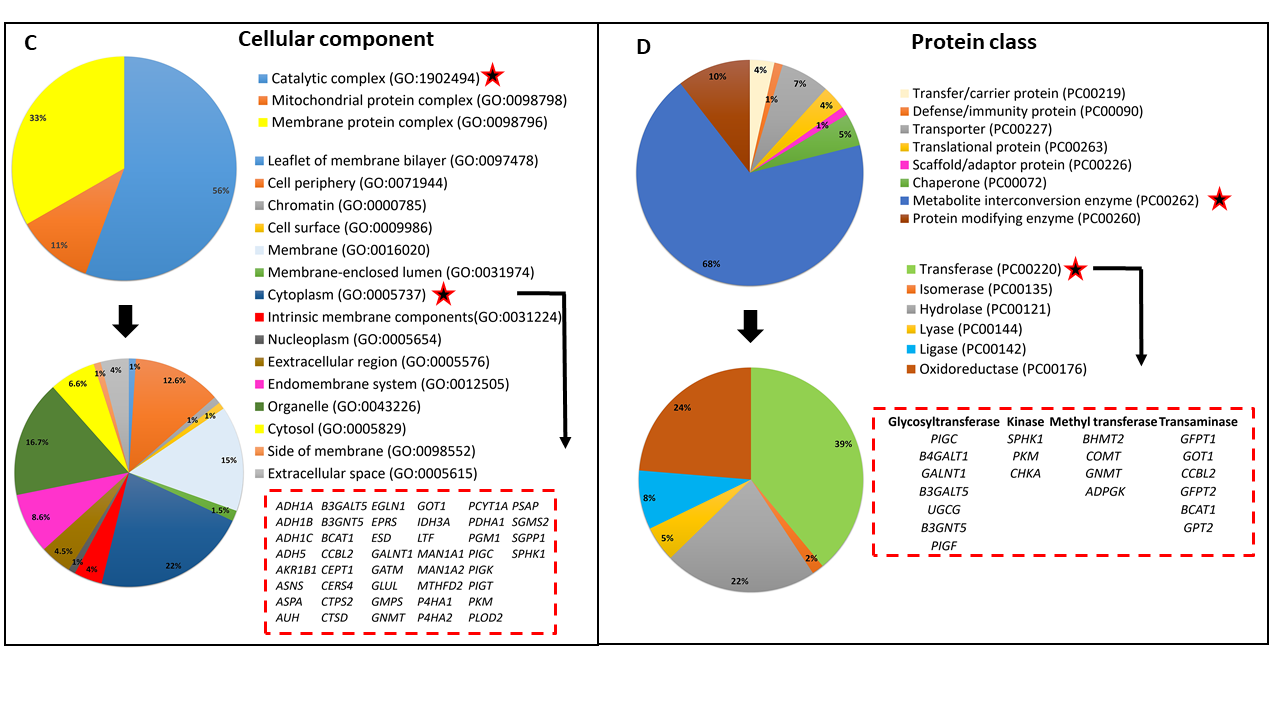


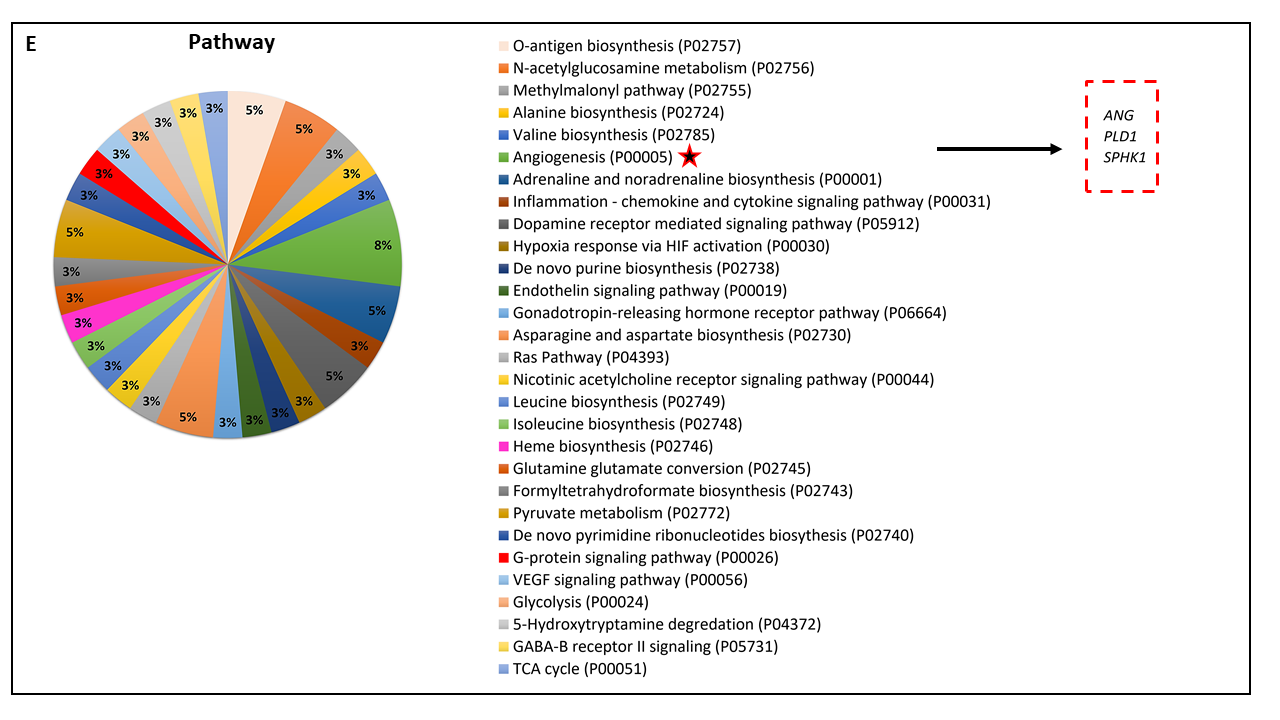


Fig. S4. Gene ontology of 102 genes that were common between CP and PDAC-CP (*A)* Molecular function of corresponding genes of altered metabolites identified in CP and PDAC and transcriptomes of CP, PDAC-CP (*B)* Biological processes associated with genes identified in CP, PDAC metabolome and CP, PDAC-CP transcriptomes (*C)* Cellular component distribution for genes identified in CP, PC metabolome and CP, PDAC-CP transcriptomes (*D)* Protein class distribution for genes identified in CP, PC metabolomics and CP, PDAC-CP transcriptomes (*E)* Pathway analysis for genes identified in CP, PC metabolomes and CP, PDAC-CP transcriptomes.

Table S1. MOOSE guidelines

|  | **MOOSE checklist** | **Brief description of how the criteria were handled in meta-analysis** |
| --- | --- | --- |
|  | **Reporting of background include** |  |
| **√** | Problem definition | Pathophysiology of Chronic pancreatitis to PDAC is unclear. |
| **√** | Hypothesis statement | We hypothesize that identifying biomarker signatures among CP and PDAC, extracting corresponding genes and integrating with transcriptome will identify therapeutic targets involved in malignant transformation in PDAC with a background of CP. |
| **√** | Description of study outcomes | Altered levels of metabolites, distribution among PDAC and CP, identification of metabolic pathways and corresponding genes |
| **√** | Type of study designs used | All platforms reporting circulatory metabolites among CP and PDAC were included; Prospective studies were selected |
| **√** | Study population | Studies included controls(healthy controls/chronic pancreatitis) and PDAC patients |
|  | **Reporting of search strategy** |  |
| **√** | Qualification of searchers | Graduate student, statistician, principal investigators, and surgical gastroenterologists. The credentials of the investigators are indicated in the author list |
| **√** | Search strategy, including time period included in the synthesis and keywords | Web of Science from March 2005 – December 2020  Scopus from March 2005 – December 2020  PubMed from March 2005 – December 2020 |
| **√** | Databases and registries | Web of science, Scopus and Pubmed |
| **√** | Search software used, name and version, including special features | Endnote was used to cross check, identify duplicate citations and merge studies |
| **√** | Use of hand searching | Manual searching did not identify additional references |
| **√** | List of citations located and excluded, including justifications | Details of literature search process are outlined in the flowchart |
| **√** | Method of addressing articles published in languages other than English | We restricted our search criteria to studies published only in English |
| **√** | Method of handling abstracts and unpublished studies | We did not include unpublished theses or abstracts |
| **√** | Description of any contact with authors | We did not contact any authors as we were able to retrieve AUC, sensitivity, patient nos., etc., from selected publications |
|  | **Reporting of methods should include** |  |
| **√** | Description of relevance or appropriateness of studies assembled for assessing hypothesis to be tested | Detailed inclusion and exclusion criteria were described in methods section |
| **√** | Rationale for the selection and coding of data | Data extracted from each of the studies were relevant to disease of interest, population characteristics, study design and outcome of interest |
| **√** | Assessment of confounding | 42% studies selected (10/24) reported sensitivity values to look at sampling designs across studies. The methodology of included studies do not address confounding |
| **√** | Assessment of study quality, including blinding of quality assessors, stratification or regression on possible predictors of study results | The selected studies AUC and computed standard error were assessed for publication bias |
| **√** | Assessment of heterogeneity | Statistical heterogeneity was tested using AUC, computed standard error between controls and samples |
| **√** | Description of statistical methods in sufficient details to be replicated | Methods of meta-analyses and sensitivity analyses are explained in the methods section |
| **√** | Provision of appropriate tables and graphics | We included 1 flow chart, 1 table of study characteristics of circulatory metabolites for 24 studies, 1 forest plot of all studies, table of sensitivity analysis and funnel plot of publication bias |
|  | **Reporting of results should include** |  |
| **√** | Graph summarizing individual study estimates and overall estimate | Fig. 2A-D |
| **√** | Table giving descriptive information for each study included | *SI Appendix* Dataset S1 |
| **√** | Results of sensitivity testing | *SI Appendix* Dataset S2 and Fig .S2 |
| **√** | Indication of statistical uncertainity of findings | 95% confidence intervals were presented for all plots |
|  | **Reporting of discussion should include** |  |
| **√** | Justification for exclusion | We excluded studies that did not report suitable control groups, studies conducted on animal tissues, cell lines and urine samples, removed duplication of data and studies pertaining to letters, reviews, theses, conference poster or proceedings |
| **√** | Assessment of quality of included studies | Selected studies reporting AUC, sensitivity and specificity |
|  | **Reporting of conclusions should include** |  |
| **√** | Consideration of alternative explanations for observed results | *SPHK1* is known to have a role in inflammatory responses, apart from tumor transformation. |
| **√** | Generalization of the conclusions | In the discussion section, we discuss the importance of *SPHK1* found upregulated after integrating corresponding genes of altered metabolites to transcriptome data in CP and PDAC-CP |
| **√** | Guidelines for future research | *SPHK1* can be tested as a therapeutic target in the progression of pancreatic cancer with underlying chronic pancreatitis. |
| **√** | Disclosure of funding source | Intramural research funding from Asian Healthcare Foundation, AIG Hospitals, Hyderabad, India |

**Table S2.** Characteristics of patients for pancreatic tissue transcriptomes

| **Particulars** | **Control pancreatic tissue** | **Chronic Pancreatitis** | **PDAC with a background of Chronic Pancreatitis** | **PDAC** |
| --- | --- | --- | --- | --- |
| **Age (years)** | 28-56 | 30 – 43 | 51 – 67 | 54 – 65 |
| **Sex** | Male | Male | Male | Male |
| **Clinical Symptoms** | Nil | Abdominal Pain  steatorrhoea | Abdominal Pain | Abdominal Pain  jaundice |
| **Imaging findings**  **EUS/ERCP** | NA | Chronic Calcific Pancreatitis with Dilated Pancreatic Duct | Pancreatic Head / Body mass with background of chronic Pancreatitis | Head Mass of Pancreas |
| **Histological examination** | Normal | Chronic Calcific Pancreatitis | Adenocarcinoma of pancreas (Body / Head mass) with background of chronic pancreatitis | Infiltrating ductal adenocarcinoma well differentiated |
| **Treatment** | Nil | Medical | Surgical – Whipple’s Pancreaticoduodenectomy | Surgical – Whipple’s Pancreaticoduodenectomy |

EUS: Endoscopic ultrasonography; ERCP: Endoscopic retrograde cholangiopancreatography

NA: not applicable; PDAC: Pancreatic ductal adenocarcinoma

Table S3. Dysregulated genes in Chronic pancreatitis(CP) and Pancreatic ductal adenocarcinoma with a background of chronic pancreatitis(PDAC-CP), tumor suppressor genes are highlighted in yellow color.

CP upregulated genes in pancreatic tissue samples

CP downregulated genes in pancreatic tissue samples

| S.No | Fold change | Gene | Name |
| --- | --- | --- | --- |
| 1 | 4.7 | *ADH1B* | All-trans-retinol dehydrogenase |
| 2 | 2.73 | *B3GNT5* | Lactosylceramide 1,3-N-acetyl-beta-D-glucosaminyltransferase |
| 3 | 2.36 | *B4GALT1* | eta-1,4-galactosyltransferase 1 |
| 4 | 2.21 | *BHMT2* | S-methylmethionine--homocysteine S-methyltransferase BHMT2 |
| 5 | 4.79 | *C1R* | Complement C1r subcomponent/(edspd1) |
| 6 | 3.5 | *CTSD* | Cathepsin D |
| 7 | 2.41 | *DDAH2* | N(G),N(G)-dimethylarginine dimethylaminohydrolase 2 |
| 8 | 3.22 | *G6PC2* | Glucose-6-phosphatase 2 |
| 9 | 3.6 | *GFPT2* | Glutamine--fructose-6-phosphate aminotransferase |
| 10 | 2.81 | *GLUL* | Glutamine synthetase |
| 11 | 2.59 | *GM2A* | Ganglioside GM2 activator |
| 12 | 2.05 | *KDM2A* | Lysine-specific demethylase 2A |
| 13 | 2.19 | *LDLR* | Sortilin-related receptor |
| 14 | 6.66 | *LTF* | Lactotransferrin |
| 15 | 5.71 | *LYZ* | Lysozyme C |
| 16 | 2.96 | *MAN1A1* | MAN1A1 |
| 17 | 2.16 | *PIGT* | GPI transamidase component PIG-T |
| 18 | 2.24 | *PLD3* | 5'-3' exonuclease PLD3 |
| 19 | 2.81 | *PPAP2B* | Phospholipid phosphatase 3 |
| 20 | 2.02 | *PSAP* | Prosaposin |
| 21 | 2.65 | *SLC7A8* | Large neutral amino acids transporter small subunit 2 |
| 22 | 2.19 | *ASPA* | Aspartoacylase |
| 23 | 2.29 | *SLCO2A1* | Solute Carrier Organic Anion Transporter Family Member 2A1 |

| S.No | Fold change | Gene | Name |
| --- | --- | --- | --- |
| 1 | -2.03 | *ACER2* | Alkaline ceramidase 2 |
| 2 | -2.09 | *B3GALT5* | Beta-1,3-galactosyltransferase 5 |
| 3 | -2.03 | *CTPS2* | CTP synthase 2 |
| 4 | -2.05 | *GLS2* | Glutaminase liver isoform, mitochondrial |
| 5 | -3.23 | *MIF* | Macrophage migration inhibitory factor |

PDAC-CP upregulated gene in pancreatic tissue

| S.No | Fold change | Gene | Name |
| --- | --- | --- | --- |
| 1 | 2.11 | *SPHK1* | Sphingosine kinase 1 |

PDAC-CP downregulated genes in pancreatic tissue

| S.No | Fold change | Gene | Name |
| --- | --- | --- | --- |
| 1 | -2.97 | *AASS* | Alpha-aminoadipic semialdehyde synthase, mitochondrial |
| 2 | -2.3 | *ADCY6* | Adenylyl cyclase type 6 |
| 3 | -2.03 | *ADH1A* | Alcohol dehydrogenase 1A |
| 4 | -4.78 | *ADH1B* | All-trans-retinol dehydrogenase [NAD(+)] ADH1B |
| 5 | -2.15 | *ADH1C* | Alcohol dehydrogenase 1C |
| 6 | -2.04 | *ADPGK* | ADP-dependent glucokinase |
| 7 | -2.76 | *AKR1B1* | Aldo-keto reductase family 1, member B1 |
| 8 | -2.78 | *AMD1* | adenosylmethionine decarboxylase 1 |
| 9 | -2.1 | *ANG* | Angiogenin |
| 10 | -2.08 | *ATP8A1* | Phospholipid-transporting ATPase IA |
| 11 | -2.79 | *AUH* | Methylglutaconyl-CoA hydratase |
| 12 | -2.28 | *CEPT1* | Choline/ethanolaminephosphotransferase 1 |
| 13 | -4.66 | *COMT* | Catechol O-methyltransferase |
| 14 | -869.27 | *CPB1* | carboxypeptidase B1 |
| 15 | -3.15 | *CPD* | Carboxypeptidase D |
| 16 | -2.3 | *GALNT1* | Polypeptide N-acetylgalactosaminyltransferase 1 |
| 17 | -2.76 | *GPCPD1* | Glycerophosphocholine phosphodiesterase |
| 18 | -2.19 | *IARS* | Isoleucine--tRNA ligase |
| 19 | -2.19 | *IDH3A* | Isocitrate dehydrogenase [NAD] subunit alpha |
| 20 | -2.67 | *KMT2C* | Histone-lysine N-methyltransferase 2C |
| 21 | -2.89 | *MAOA* | Amine oxidase [flavin-containing] A |
| 22 | -2.1 | *PCYT1A* | Choline-phosphate cytidylyltransferase A |
| 23 | -3.69 | *PDE5A* | PDE5A |
| 24 | -2.6 | *PIGK* | GPI-anchor transamidase |
| 25 | -2.16 | *PLD1* | Phospholipase D1 |
| 26 | -3.06 | *PLOD2* | Procollagen-lysine,2-oxoglutarate 5-dioxygenase 2 |
| 27 | -3 | *PPAP2A* | Phospholipid phosphatase 1 |
| 28 | -2.34 | *PPIF* | Peptidyl-prolyl cis-trans isomerase F, mitochondrial |
| 29 | -3.06 | *PPIG* | Peptidyl-prolyl cis-trans isomerase G |
| 30 | -2.13 | *SLC10A1* | Solute Carrier Family 10 Member 1 |
| 31 | -2.18 | *SGMS2* | Phosphatidylcholine:ceramide cholinephosphotransferase 2 |
| 32 | -2.74 | *UGCG* | Ceramide glucosyltransferase |

Common upregulated genes identified among CP and PDAC-CP in pancreatic tissue samples

| S.No | CP Fold change | PDAC-CP  Fold change | Gene | Name |
| --- | --- | --- | --- | --- |
| 1 | 2.57 | 2.46 | *PIGC* | Phosphatidylinositol N-acetylglucosaminyltransferase subunit C |
| 2 | 4.62 | 2.94 | *PKM* | Pyruvate Kinase M1/2 |
| 3 | 3.24 | 2.43 | *PPIB* | Peptidyl-prolyl cis-trans isomerase B |

Common downregulated genes identified among CP and PDAC-CP in pancreatic tissue samples

| S.No | CP Fold change | PDAC-CP  Fold change | Gene | Name |
| --- | --- | --- | --- | --- |
| 1 | -2.18 | -3.34 | *ADH5* | Alcohol dehydrogenase class-3 |
| 2 | -2.67 | -3.97 | *ASNS* | Asparagine synthetase |
| 3 | -2.51 | -20.54 | *AZGP1* | Zinc-alpha-2-glycoprotein |
| 4 | -4.15 | -3.33 | *BCAT1* | Branched-chain-amino-acid aminotransferase, cytosolic |
| 5 | -4.09 | -4.39 | *CCBL2* | cysteine conjugate-beta lyase 2 |
| 6 | -2.11 | -2.18 | *CERS4* | Ceramide synthase 4 |
| 7 | -2.13 | -2.45 | *CHKA* | Choline kinase alpha |
| 8 | -2.09 | -2.45 | *CYP51A1* | cytochrome P450, family 51, subfamily A, polypeptide 1 |
| 9 | -2.66 | -3.99 | *DLD* | Dihydrolipoamide dehydrogenase |
| 10 | -3.92 | -9.08 | *EGLN1* | Egl nine homolog 1 |
| 11 | -2.21 | -2.82 | *EPRS* | Bifunctional glutamate/proline--tRNA ligase |
| 12 | -11.73 | -14.81 | *ESD* | S-formylglutathione hydrolase |
| 13 | -145.43 | -919.44 | *GATM* | Glycine amidinotransferase, mitochondrial |
| 14 | -3.16 | -5.72 | *GFPT1* | Glutamine--fructose-6-phosphate aminotransferase |
| 15 | -3.12 | -4.05 | *GLS* | Glutaminase kidney isoform, mitochondrial |
| 16 | -2.13 | -2.33 | *GMPS* | GMP synthase |
| 17 | -32.06 | -39.44 | *GNMT* | Glycine N-methyltransferase |
| 18 | -2.5 | -2.97 | *GOT1* | Aspartate aminotransferase |
| 19 | -6 | -5.53 | *GPT2* | Alanine aminotransferase 2 |
| 20 | -2.61 | -3.79 | *LARS* | Leucine--tRNA ligase |
| 21 | -5.16 | -7.67 | *LDHA* | L-lactate dehydrogenase A chain |
| 22 | -7.36 | -11.76 | *LDHB* | L-lactate dehydrogenase B chain |
| 23 | -3.86 | -5.5 | *MAN1A2* | Mannosyl-oligosaccharide 1,2-alpha-mannosidase IB |
| 24 | -2.21 | -2.82 | *MAN2A1* | Mannosyl-oligosaccharide 1,2-alpha-mannosidase IB |
| 25 | -2.29 | -2.52 | *MCCC1* | Methylcrotonoyl-CoA carboxylase subunit alpha, mitochondrial |
| 26 | -5.66 | -6.28 | *MTHFD2* | Bifunctional methylenetetrahydrofolate dehydrogenase/cyclohydrolase |
| 27 | -7.11 | -6.66 | *NSMAF* | Protein FAN |
| 28 | -2.18 | -2.29 | *P4HA1* | Prolyl 4-hydroxylase subunit alpha-1 |
| 29 | -2.39 | -2.74 | *P4HA2* | Prolyl 4-hydroxylase subunit alpha-2 |
| 30 | -2.52 | -4.43 | *PDHA1* | Pyruvate dehydrogenase alpha 1 |
| 31 | -3.45 | -3.6 | *PGM1* | Phosphoglucomutase-1 |
| 32 | -3.36 | -2.8 | *PIGF* | Phosphatidylinositol-glycan biosynthesis class F protein |
| 33 | -3 | -225.18 | *PLA2G1B* | Phospholipase A2 |
| 34 | -52.76 | -79.95 | *PPIA* | Peptidyl-prolyl cis-trans isomerase A |
| 35 | -4.1 | -52.98 | *RNASE1* | Ribonuclease A Family Member 1, Pancreatic |
| 36 | -3.02 | -2.97 | *SGPP1* | Sphingosine-1-phosphate phosphatase 1 |
| 37 | -2.18 | -2.4 | *SLC16A7* | Monocarboxylate transporter 2 |
| 38 | -2.56 | -3.13 | *SLC44A3* | Choline transporter-like protein 3 |

**Table S4.** Dysregulated genes in Pancreatic ductal adenocarcinoma with a background of chronic pancreatitis(PDAC-CP) and PDAC, tumor suppressor genes are highlighted in yellow color

PDAC-CP upregulated gene in pancreatic tissue samples

| S.No | Fold  change | PDAC-CP | Name |
| --- | --- | --- | --- |
| 1 | 2.11 | *SPHK1* | **Sphingosine kinase 1** |

PDAC-CP downregulated genes in pancreatic tissue samples

| S.No | Fold change | Gene | Name |
| --- | --- | --- | --- |
| 1 | -2.97 | *AASS* | Alpha-aminoadipic semialdehyde synthase, mitochondrial |
| 2 | -2.3 | *ADCY6* | Adenylate cyclase type 6 |
| 3 | -2.03 | *ADH1A* | Alcohol dehydrogenase 1A |
| 4 | -2.15 | *ADH1C* | Alcohol dehydrogenase 1C |
| 5 | -2.04 | *ADPGK* | ADP-dependent glucokinase |
| 6 | -2.76 | *AKR1B1* | Aldo-keto reductase family 1 member B1 |
| 7 | -2.1 | *ANG* | Angiogenin |
| 8 | -2.28 | *CEPT1* | Choline/ethanolaminephosphotransferase 1 |
| 9 | -4.66 | *COMT* | Catechol O-methyltransferase |
| 10 | -3.15 | *CPD* | Carboxypeptidase D |
| 11 | -2.3 | *GALNT1* | Polypeptide N-acetylgalactosaminyltransferase 1 |
| 12 | -2.76 | *GPCPD1* | Glycerophosphocholine phosphodiesterase |
| 13 | -2.19 | *IARS* | Isoleucine--tRNA ligase |
| 14 | -2.19 | *IDH3A* | Isocitrate dehydrogenase [NAD] subunit alpha |
| 15 | -2.67 | *KMT2C* | Histone-lysine N-methyltransferase 2C |
| 16 | -2.1 | *PCYT1A* | Choline-phosphate cytidylyltransferase A |
| 17 | -3.69 | *PDE5A* | cGMP-specific 3',5'-cyclic phosphodiesterase |
| 18 | -2.6 | *PIGK* | GPI-anchor transamidase |
| 19 | -2.16 | *PLD1* | Phospholipase D1 |
| 20 | -3.06 | *PLOD2* | Procollagen-lysine,2-oxoglutarate 5-dioxygenase 2 |
| 21 | -2.34 | *PPIF* | Peptidyl-prolyl cis-trans isomerase F, mitochondrial |
| 22 | -2.18 | *SGMS2* | Phosphatidylcholine:ceramide cholinephosphotransferase 2 |
| 23 | -2.13 | *SLC10A1* | Sodium/bile acid cotransporter |
| 24 | -2.74 | *UGCG* | Ceramide glucosyltransferase |

PDAC upregulated genes in pancreatic tissue samples

| S.No | Fold change | Gene | Name |
| --- | --- | --- | --- |
| 1 | 3.07 | *ABCA7* | Phospholipid-transporting ATPase ABCA7 |
| 2 | 2.91 | *ABCC3* | ATP-binding cassette sub-family C member 3 |
| 3 | 2.3 | *ACER3* | Alkaline ceramidase 3 |
| 4 | 2.54 | *ADPGK* | ADP-dependent glucokinase |
| 5 | 2.32 | *ALG3* | Dol-P-Man:Man(5)GlcNAc(2)-PP-Dol alpha-1,3-mannosyltransferase |
| 6 | 2.07 | *ASAH1* | Acid ceramidase |
| 7 | 3.97 | *B3GALT5* | Beta-1,3-galactosyltransferase 5 |
| 8 | 3.85 | *B3GNT5* | Lactosylceramide 1,3-N-acetyl-beta-D-glucosaminyltransferase |
| 9 | 2.53 | *CKM* | Creatine kinase M-type |
| 10 | 2.37 | *CKMT1A* | Creatine kinase U-type, mitochondrial |
| 11 | 2.2 | *CKMT1B* | Creatine kinase U-type, mitochondrial |
| 12 | 2.03 | *CPT1A* | Carnitine O-palmitoyltransferase 1, liver isoform |
| 13 | 2.1 | *CPT1C* | Carnitine O-palmitoyltransferase 1, brain isoform |
| 14 | 5.44 | *CTSD* | Cathepsin D |
| 15 | 2.93 | *CYP1B1* | Cytochrome P450 1B1 |
| 16 | 2.24 | *DDAH2* | N(G),N(G)-dimethylarginine dimethylaminohydrolase 2 |
| 17 | 2.03 | *DGKD* | Diacylglycerol kinase delta |
| 18 | 2.88 | *DOT1L* | Histone-lysine N-methyltransferase, H3 lysine-79 specific |
| 19 | 2.25 | *EGLN2* | Prolyl hydroxylase EGLN2 |
| 20 | 4.32 | *GFPT2* | Glutamine--fructose-6-phosphate aminotransferase [isomerizing] 2 |
| 21 | 2.21 | *GLTPD2* | Glycolipid transfer protein domain-containing protein 2 |
| 22 | 5.22 | *GM2A* | Ganglioside GM2 activator |
| 23 | 2.09 | *GNPTAB* | N-acetylglucosamine-1-phosphotransferase subunits alpha/beta |
| 24 | 2.18 | *GPI* | Glucose-6-Phosphate Isomerase |
| 25 | 2.1 | *GZMB* | Granzyme B |
| 26 | 3.52 | *HK1* | Hexokinase-1 |
| 27 | 2.35 | *HK2* | Hexokinase-2 |
| 28 | 2.13 | *KARS* | Lysine--tRNA ligase |
| 29 | 2.31 | *KDM2A* | Lysine-specific demethylase 2A |
| 30 | 4.26 | *KMT2A* | Histone-lysine N-methyltransferase 2A |
| 31 | 3.06 | *KYNU* | Kynureninase |
| 32 | 2.11 | *L3HYPDH* | Trans-3-hydroxy-L-proline dehydratase |
| 33 | 2.62 | *LYZ* | Lysozyme C |
| 34 | 2.33 | *MAN2B1* | Lysosomal alpha-mannosidase |
| 35 | 2.47 | *NPL* | Nuclear protein localization protein 4 homolog |
| 36 | 2.22 | *NSD1* | Histone-lysine N-methyltransferase, H3 lysine-36 specific |
| 37 | 2.12 | *P4HA3* | Prolyl 4-hydroxylase subunit alpha-3 |
| 38 | 2.11 | *PIGT* | GPI transamidase component PIG-T |
| 39 | 2.16 | *PISD* | Phosphatidylserine decarboxylase proenzyme, mitochondrial |
| 40 | 2.21 | *PLD3* | 5'-3' exonuclease PLD3 |
| 41 | 3.13 | *PLOD1* | Procollagen-lysine,2-oxoglutarate 5-dioxygenase 1 |
| 42 | 2.84 | *PPIH* | Peptidyl-prolyl cis-trans isomerase H |
| 43 | 2.53 | *PRRX1* | Paired mesoderm homeobox protein 1 |
| 44 | 2.29 | *QARS* | Glutamine--tRNA ligase |
| 45 | 2.76 | *SGMS2* | Phosphatidylcholine:ceramide cholinephosphotransferase 2 |
| 46 | 4.6 | *SGPL1* | Sphingosine-1-phosphate lyase 1 |
| 47 | 2.54 | *SLC16A1* | Monocarboxylate transporter 1 |
| 48 | 2.88 | *SLC16A3* | Monocarboxylate transporter 4 |
| 49 | 2.51 | *SLC16A4* | Monocarboxylate transporter 5 |
| 50 | 2.9 | *SLC2A1* | Solute carrier family 2, facilitated glucose transporter member 1 |
| 51 | 2.77 | *SLC36A1* | Proton-coupled amino acid transporter 1 |
| 52 | 3.72 | *SLC44A4* | Choline transporter-like protein 4 |
| 53 | 14.33 | *SLC6A14* | Sodium- and chloride-dependent neutral and basic amino acid transporter B |
| 54 | 2.27 | *SLC6A8* | Sodium- and chloride-dependent creatine transporter 1 |
| 55 | 2.35 | *SLC7A8* | Large neutral amino acids transporter small subunit 2 |
| 56 | 2.34 | *SLCO2B1* | Solute carrier organic anion transporter family member 2B1 |
| 57 | 2.18 | *SMPD3* | Sphingomyelin phosphodiesterase 3 |
| 58 | 2.31 | *SRR* | Serine racemase |
| 59 | 3.45 | *TGM2* | Protein-glutamine gamma-glutamyltransferase 2 |
| 60 | 2.06 | *TREH* | Trehalase |
| 61 | 2.22 | *WBSCR22* | Probable 18S rRNA (guanine-N(7))-methyltransferase |
| 62 | 2.35 | *WHSC1L1* | Histone-lysine N-methyltransferase NSD3 |

PDAC downregulated genes in pancreatic tissue samples

| S.No | Fold change | Gene | Name |
| --- | --- | --- | --- |
| 1 | -2.99 | *ASNS* | Asparagine synthetase [glutamine-hydrolyzing] |
| 2 | -16.87 | *AZGP1* | Zinc-alpha-2-glycoprotein |
| 3 | -3.27 | *BCAT1* | Branched-chain-amino-acid aminotransferase, cytosolic |
| 4 | -4.2 | *CCBL2* | Kynurenine--oxoglutarate transaminase 3 |
| 6 | -2.94 | *EGLN1* | Egl nine homolog 1 |
| 7 | -9.72 | *ESD* | S-formylglutathione hydrolase |
| 8 | -951.44 | *GATM* | Glycine amidinotransferase, mitochondrial |
| 9 | -2.87 | *GLS* | Glutaminase kidney isoform, mitochondrial |
| 10 | -31.55 | *GNMT* | Glycine N-methyltransferase |
| 11 | -5.89 | *GPT2* | Alanine aminotransferase 2 |
| 12 | -2.05 | *LARS* | Leucine--tRNA ligase, cytoplasmic |
| 13 | -4.13 | *LDHA* | L-lactate dehydrogenase A chain |
| 14 | -16.36 | *LDHB* | L-lactate dehydrogenase B chain |
| 15 | -3.54 | *MAN1A2* | Mannosyl-oligosaccharide 1,2-alpha-mannosidase IB |
| 16 | -2.03 | *MCCC1* | Methylcrotonoyl-CoA carboxylase subunit alpha, mitochondrial |
| 17 | -5.64 | *MIF* | Macrophage migration inhibitory factor |
| 18 | -3.53 | *MTHFD2* | Bifunctional methylenetetrahydrofolate dehydrogenase/cyclohydrolase, mitochondrial |
| 19 | -7.1 | *NSMAF* | Protein FAN |
| 20 | -2.09 | *PDHA1* | Pyruvate dehydrogenase E1 component subunit alpha, somatic form, mitochondrial |
| 21 | -2.32 | *PGM1* | Phosphoglucomutase-1 |
| 22 | -2.25 | *PIGF* | Phosphatidylinositol-glycan biosynthesis class F protein |
| 23 | -2.08 | *PIGG* | GPI ethanolamine phosphate transferase 2 |
| 24 | -2573.77 | *PLA2G1B* | Phospholipase A2 |
| 25 | -23.53 | *PPIA* | Peptidyl-prolyl cis-trans isomerase A |
| 26 | -3.05 | *PROSC* | Pyridoxal phosphate homeostasis protein |
| 27 | -49.02 | *RNASE1* | Ribonuclease pancreatic |
| 28 | -3.06 | *SGPP1* | Sphingosine-1-phosphate phosphatase 1 |
| 29 | -2.49 | *SLC44A3* | Choline transporter-like protein 3 |

Common upregulated genes identified among PDAC-CP and PDAC in pancreatic tissue samples

| S.No | PDAC-CP  Fold change | PDAC  Fold change | Gene | Name |
| --- | --- | --- | --- | --- |
| 1 | 2.46 | 6.78 | PIGC | Phosphatidylinositol N-acetylglucosaminyltransferase subunit C |
| 2 | 2.94 | 5.41 | *PKM* | Pyruvate Kinase M1/2 |
| 3 | 2.43 | 3.81 | *PPIB* | Peptidyl-prolyl cis-trans isomerase B |

Common downregulated genes identified among PDAC-CP and PDAC in pancreatic tissue samples

| S.No | PDAC-CP  Fold change | PDAC  Fold change | Gene | Name |
| --- | --- | --- | --- | --- |
| 1 | -4.78 | -2.21 | *ADH1B* | All-trans-retinol dehydrogenase [NAD(+)] ADH1B |
| 2 | -2.78 | -2.46 | *AMD1* | S-adenosylmethionine decarboxylase proenzyme |
| 3 | -2.08 | -2.47 | *ATP8A1* | Phospholipid-transporting ATPase IA |
| 4 | -2.79 | -2.16 | *AUH* | Methylglutaconyl-CoA hydratase, mitochondrial |
| 5 | -869.27 | -1701.23 | *CPB1* | Carboxypeptidase B |
| 6 | -2.89 | -2.32 | *MAOA* | Amine oxidase [flavin-containing] A |
| 7 | -3 | -2.47 | *PPAP2A* | Phospholipid phosphatase 1 |
| 8 | -3.06 | -2.12 | *PPIG* | Peptidyl-prolyl cis-trans isomerase G |

**Table S5. Fisher’s exact test between CP, PDAC-CP and PC groups.**

Comparison of CP, PDAC-CP; PDAC-CP, PDAC groups for vessel, acini, duct and islet tissues

| **S. No** | **Group** | **Tissue** | **High positives** | **Low positives and positives** | **P-Value** |
| --- | --- | --- | --- | --- | --- |
| 1 | CP | Vessel | 1/13 | 8/15 | 0.015 |
|  | PDAC-CP |  | 12/13 | 7/15 |  |
| 2 | PDAC-CP | Vessel | 12/14 | 7/11 | 0.350 |
|  | PDAC |  | 2/14 | 4/11 |  |
| 3 | CP | Acini | 8/8 | 5/18 | 0.001 |
|  | PDAC-CP |  | 0 | 13/18 |  |
| 4 | PDAC-CP | Acini | 0 | 13/26 | NA |
|  | PDAC |  | 0 | 13/26 |  |
| 5 | CP | Duct | 6/12 | 2/9 | 0.366 |
|  | PDAC-CP |  | 6/12 | 7/9 |  |
| 6 | PDAC-CP | Duct | 6/6 | 7/19 | 0.014 |
|  | PDAC |  | 0 | 12/19 |  |
| 7 | CP | Islet | 10/11 | 6/11 | 0.148 |
|  | PDAC-CP |  | 1/11 | 5/11 |  |
| 8 | PDAC-CP | Islet | 1/2 | 5/12 | 1.0 |
|  | PDAC |  | 1/2 | 7/12 |  |

Dataset S1. Circulatory metabolites identified in chronic pancreatitis, pancreatic cancer and pancreatic ductal adenocarcinoma using metabolomics platforms

**Dataset S2**. Publication bias funnel plot, sensitivity forest plot among the studies reporting circulatory metabolites

**Dataset S3.** Metabolites list derived from metabolomics studies comparing healthy controls and pancreatic cancer/PDAC; healthy controls/chronic pancreatitis and pancreatic cancer/PDAC; chronic pancreatitis and pancreatic cancer/PDAC

**Dataset S4.** List of genes derived from relational databases

**Dataset S5.** Splicing index of dysregulated genes
